# Gene dosage compensation of rRNA transcript levels in *Arabidopsis thaliana* lines with reduced ribosomal gene copy number

**DOI:** 10.1093/plcell/koab020

**Published:** 2021-02-02

**Authors:** Francesca B Lopez, Antoine Fort, Luca Tadini, Aline V Probst, Marcus McHale, James Friel, Peter Ryder, Fr�d�ric Pontvianne, Paolo Pesaresi, Ronan Sulpice, Peter McKeown, Galina Brychkova, Charles Spillane

**Affiliations:** 1 Genetics and Biotechnology Laboratory, Plant & AgriBiosciences Research Centre (PABC), Ryan Institute, National University of Ireland Galway, Galway H91 REW4, Ireland; 2 Systems Biology Laboratory, Plant & AgriBiosciences Research Centre (PABC), Ryan Institute, National University of Ireland Galway, Galway H91 REW4, Ireland; 3 Dipartimento di Bioscienze, Universit� degli Studi di Milano, 20133 Milano, Italy; 4 CNRS, GReD, Universit� Clermont Auvergne, INSERM, 63001 Clermont–Ferrand, France; 5 CNRS, Laboratoire G�nome et D�veloppement des Plantes (LGDP), Universit� de Perpignan Via Domitia, Perpignan, France

## Abstract

The 45S rRNA genes (rDNA) are among the largest repetitive elements in eukaryotic genomes. rDNA consists of tandem arrays of rRNA genes, many of which are transcriptionally silenced. Silent rDNA repeats may act as ‘back-up’ copies for ribosome biogenesis and have nuclear organization roles. Through Cas9-mediated genome editing in the *Arabidopsis thaliana* female gametophyte, we reduced 45S rDNA copy number (CN) to a plateau of ∼10%. Two independent lines had rDNA CNs reduced by up to 90% at the T7 generation, named low copy number (LCN) lines. Despite drastic reduction of rDNA copies, rRNA transcriptional rates, and steady-state levels remained the same as wild-type plants. Gene dosage compensation of rRNA transcript levels was associated with reduction of silencing histone marks at rDNA loci and altered Nucleolar Organiser Region 2 organization. Although overall genome integrity of LCN lines appears unaffected, a chromosome segmental duplication occurred in one of the lines. Transcriptome analysis of LCN seedlings identified several shared dysregulated genes and pathways in both independent lines. Cas9 genome editing of rRNA repeats to generate LCN lines provides a powerful technique to elucidate rDNA dosage compensation mechanisms and impacts of low rDNA CN on genome stability, development, and cellular processes.

## Teaser

Ribosomal gene reduction does not affect rRNA homeostasis but is correlated with loss of enrichment of heterochromatic marks, signs of genome instability and dysregulated metabolic pathways.

## Introduction

Ribosomal RNA (rRNA) genes are one of the most abundant repetitive elements in eukaryotic genomes and are typically clustered as tandem repeats containing hundreds or thousands of rRNA gene copies, known as rDNA, and usually associated with the nucleolus. The 45S rDNA loci consist of sections encoding the 18S, 5.8S, and 25S/28S rRNAs, which comprise the catalytic components of the mature ribosome, two Internal Transcribed Spacers, as well as two external transcribed spacers (S�ez-V�squez and Delseny, 2019). The 45S arrays are associated with large chromosomal regions known as Nucleolar Organiser Regions (NORs): when stained with silver nitrate the NORs appear as dark areas on metaphase chromosomes and can be observed forming the nucleolus during interphase (McClintock, 1934).

To meet cellular demand for production of rRNA during cell proliferation, eukaryotes have a dedicated transcription system, RNA polymerase I (Pol I) and highly duplicated 45S rDNA regions which provides hundreds or thousands of 45S rDNA copies to transcribe. The transcription of 45S rRNA can comprise in excess of 80% of total cellular transcription during proliferation. The epigenetic mechanisms which orchestrate the activation or silencing of rRNA genes have been widely documented in a range of model organisms (Grummt and Pikaard, 2003; McStay and Grummt, 2008). For instance, the *Saccharomyces cerevisiae* genome features approximately 150 rRNA copies on chromosome XII which are regulated by a mechanism which has likely evolved to stably maintain the number of rDNA copies in the genome (Kobayashi, 2006). Notably, silenced 45S rDNA copies are involved in maintenance of genome stability and cell senescence (Kobayashi, 2014), highlighting their functional role(s) in the maintenance of cellular homeostasis. It has been demonstrated that yeast cells with 80% reduction in rDNA copies suffer from increased DNA damage due to a reduced ability to repair Double Strand Breaks (DSB) (Ide et�al., 2010). Similarly, in mammals a role for silent rDNA copies in the maintenance of genomic stability is now well established (Stochaj and Weber, 2020), where the reduction of 45S rDNA copy number (CN) (often coupled with amplification of 5S rDNA loci) has been associated with the onset of a range of cancers (Xu et�al., 2017; Udugama et�al., 2018).

Plants can exhibit extensive copy number variation (CNV) at their 45S rDNA loci. For instance, in inbred wild accessions of *A. thaliana* sourced from across Sweden, the 45S rDNA CN ranged from 500 to 2500, with rDNA CN strongly correlating with genome size (Long et�al., 2013). In *A. thaliana*, the rDNA arrays are located on the acrocentric chromosomes 2 and 4 (Copenhaver and Pikaard, 1996), adjacent to the telomeric repeats. Remarkably, even closely related accessions of *A. thaliana* can display considerable 45S rDNA CNV, which has been predominantly attributed to variation in 45S rDNA repeats on chromosome 2 (NOR2) (Rabanal et�al., 2017). The same study reported that 45S rDNA CNV can also be found within isogenic lines of the same accession, as well as in recombinant inbred lines. The underlying molecular basis for such variation appears to be due to crossing-over events, rather than the activity of homologous recombination (HR). In support of this, Sims et al., found that rDNA loci are shielded from HR components during meiosis, likely as a mechanism to avoid deleterious non-allelic interactions (Sims et�al., 2019).

In multicellular eukaryotes, the transcriptional silencing of rRNA occurs during development, a phenomenon requiring the reorganization of chromatin, and DNA methylation patterns across megabase tracts of chromosomes. The mechanisms to achieve multimegabase silencing which have evolved at cellular and organismal levels are diverse among different model organisms (Bersaglieri and Santoro, 2019). For instance, as mammalian cells exit a pluripotent state and begin differentiating, the silencing of rDNA copies is established *de novo* by the nucleolar remodeling complex (NoRC) (Santoro et�al., 2002) through cytosine methylation of the rDNA promoter, accompanied by tight nucleosomal packaging of the rDNA coding sequence (associated with enrichments in silencing histone marks such as H3K9me2).

Nucleolar dominance was initially observed by Navashin as secondary constrictions in chromosomes of F1 progeny of interspecific hybrids, where the constriction only occurred on the chromosomes inherited from one of the parents (Navashin, 1934). Nucleolar dominance was later shown in Xenopus hybrids to be due to the uniparental expression of rDNA loci (Honjo and Reeder, 1973). In plants, many epigenetic mechanisms that regulate rRNA transcription in the context of nucleolar dominance have been studied (Chen and Pikaard, 1997; Lawrence et�al., 2004), where the epigenetic mechanisms which maintain rDNA silencing in nucleolar dominance appear to also control silencing of rDNA during development. Unlike in mammals, such transcriptional silencing of rDNA loci is not dependent on the NoRC, but instead achieved by the concerted action of a number of chromatin remodeling factors (particularly HISTONE DEACETYLASE6), both cytosine and histone methyltransferases, as well as methylcytosine binding domain proteins (MBD6 and MBD10) which together mediate the large-scale silencing of rRNA genes (Probst et�al., 2004; Preuss et�al., 2008; Tucker et�al., 2010; Pontvianne et�al., 2012).

In the *A. thaliana* accession Col-0 (which harbors approximately 375 45S rDNA copies per NOR silencing has been characterized as a chromosomal position-dependent phenomenon, where NOR2 is developmentally silenced 10–20 days post germination, while NOR4 remains available for transcription throughout vegetative development, thus leaving about 50% of 45S rDNA copies competent for transcription (Pontvianne et�al., 2010; Mohannath et�al., 2016). As a result, in plants, as in animals, rRNA genes can be classified as active, inactive, or silent depending on their chromatin organization (McKeown and Shaw, 2009). Ascribing any functional role of rDNA CNV in plants has remained elusive due to the lack of molecular tools to elicit a targeted reduction in rDNA CN.

However, in *A. thaliana* reduction of rDNA CN has been reported in loss-of-function mutants of two of the large subunits of CHROMATIN ASSEMBLY FACTOR1 (CAF-1) (Mozgova et�al., 2010). The CAF-1 complex is necessary for H3–H4 deposition and chromatin assembly following DNA replication: it is formed of three protein subunits, FASCIATA1 (FAS1), FASCIATA2 (FAS2), and MULTICOPY SUPPRESSOR OF IRA1 (MSI1). The *fas1 fas2* double mutant displays progressive transgenerational shortening of telomeres on all chromosome arms, which is further associated with loss of 45S rRNA CN on NOR2 and NOR4 (Pontvianne et�al., 2013). The phenotypes of the *fas1 fas2* double mutants are lost when wild-type (WT) alleles are reintroduced, whether at early or late generations (Pavlištov� et�al., 2016). Genetic complementation approaches of the *fas1 fas2* mutant have generated a *FAS1 FAS2* complemented line which features a loss of up to 80% of 45S rDNA copies, but reported to resemble the WT phenotype (Pavlištov� et�al., 2016).

Here, we use CRISPR-Cas9-induced DSBs at 45S rDNA loci that can be used to generate plant lines with altered 45S rDNA CN. To identify the lower limits of 45S rDNA CN which still allow for viable plants, over six generations, we generated lines with up to 93% reduction in 45S rDNA CN. We have also investigated how rRNA transcript rates can be maintained despite such drastic reductions in 45S rDNA CN and demonstrate that dosage compensation of rRNA production appears to be achieved through chromatin re-organization at the rDNA loci, rather than through alterations of the transcription steady state. Using Nanopore genome sequencing, we further screened for genomic alterations in two independent 45s rDNA low copy number (LCN) lines to determine whether genome stability could be compromised by loss of 45S rDNA CN. CRISPR-Cas9-induced deletion of copies from tandem repeat regions, such as the 45s rDNA in plants, provides a new tool to understand the roles of such loci.

## Results

### Cas9-induced DSBs at the 18S loci cause reduction of 45S rDNA CN

To determine the impact of reducing rDNA levels to their functional minimum in a model plant, we reduced the number of 45S rDNA copies in *A. thaliana* using transgenerational Cas9 targeting of the 45S rDNA repeats (Figure�1). To achieve such CN reductions, we designed a single guide RNA (gRNA) specific to the 18S locus within the 45S rDNA repeats (with no predicted off-target site) using the CRISPR-P online tool (http://cbi.hzau.edu.cn/crispr/). Using a previously described vector (Wang et�al., 2015; Ryder et�al., 2017), we developed a transgene cassette (pHEE-18S) containing the 18S gRNA. This transgene cassette allows expression of Cas9 exclusively in the egg cell (EC) of the haploid female gametophyte, where we hypothesized that Cas9 activity across the 45S rDNA repeats would generate either large deletions or insertions of the repeats, through the subsequent activity of the error-prone non-homologous end joining DNA repair pathway (Figure�1C) (Cubbon et�al., 2018). Spatiotemporally localizing Cas9 expression to the EC of the female gametophyte also allowed us to investigate the effects of CN mutagenesis in the absence of Cas9 activity during other crucial stages of the life cycle such as meiosis, fertilization and seed development. The T1 transformant seedlings were sown on hygromycin selective media and genotyped for 45S rDNA CN by qPCR.

**Figure 1:**
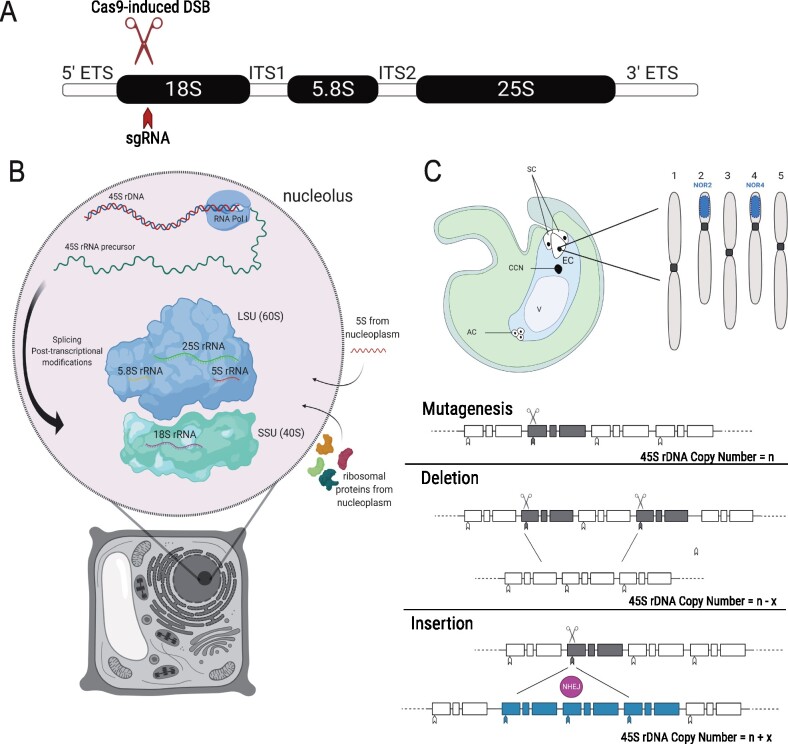
Overview of 45S rDNA loci, dynamics of transcription and ribosome assembly, and mutagenesis concept. A, The 45S rRNA gene comprises two ETSs, two ITSs, and the 18S, 5.8S, and 25S (28S in fungi and animals) ribosomal components. A single gRNA (sgRNA) was designed specifically to guide Cas9 to the 18S locus and generate DSBs across the 45S repeats. B, Transcription of 45S rRNA occurs in the nucleolus, the largest structure in the nucleus. Assembly of ribosomes requires import of the 5S transcript from the nucleoplasm, as well as import of ribosomal proteins into the nucleolus. LSU = Large Subunit; SSU = Small Subunit. C, CRISPR-Cas9 mutagenesis: Random DSBs (gray) were generated along the 45S repeats targeting the 18S locus within the 45S rRNA gene. Two outcomes were expected: deletions of large numbers of repeats or insertion of supernumerary copies (blue) by the action of NHEJ DNA repair machinery. AC, Antipodal cells; V, Vacuole; CCN, Central cell nucleus; and SC, Synergid cells.

We recovered a population of T1 plants displaying large CNV in the 45S rDNA (Figure�2A), ranging from ∼20% to 160% CN compared with WT. Whilst selection was initially performed to identify lines with CN loss (e.g. ∼20% of WT copies, line #236, and #289, Figure�2A) and CN gain, we determined that Cas9 activity predominantly causes transgenerational reduction of 45S CN. Hence a fixed increase in CN of 45S repeats could not be maintained over successive generations.

**Figure 2:**
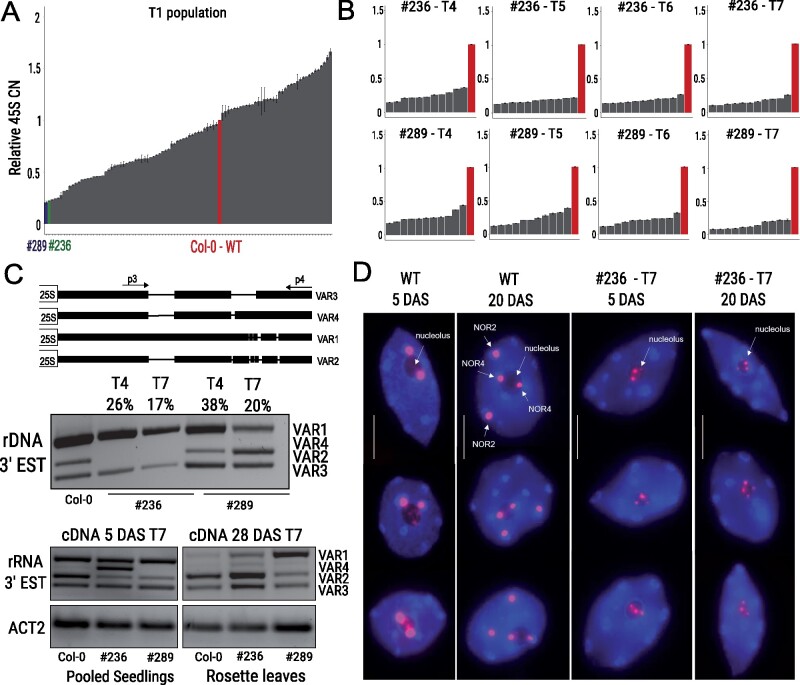
Selection of Low 45S CN lines, 3'-ETS variant analysis and FISH at T7 generation. A, 45S rDNA Relative CN of T1 transformant population quantified by qPCR against the single copy genes *HXK1* and *TZF1*. Blue bar and green bar indicate lines #289 and #236, respectively. Highlighted are LCN lines on which all experiments were performed. B, 45S rDNA Relative CN by quantification by qPCR of T4 to T7 generations. Eleven individual plants of lines #236 and #289 were selected per generation and one WT Col-0 control. Bars represent standard error of technical qPCR replicates. C, Schematic representation of 3′-allelic variants, adapted from Pontvianne, 2010. Analysis of genomic and gene dosage of 45S rDNA variants in WT and LCN at T4 and T7 generations. Percentage (%) of 45S relative CN for each was calculated by qPCR using the same DNA sample as the RT-PCR. RT-PCR analysis of 3′-EST variant expression shows qualitative differences between WT and LCN lines, indicating mutagenesis causes qualitative differences in variant expression. 45S relative CN was calculated by qPCR as described. D, Representative nuclei subjected to FISH for 45S rDNA (red) from whole cotyledon and leaf tissues in WT and line #236 (T7). DNA is counterstained with DAPI (blue). In WT seedlings, both NORs localize at the nucleolus. The diffused signal within the nucleolus suggests chromatin de-condensation. After approximately 15 DAS, NOR2 is progressively silenced and moves away from the nucleolus. NOR4 localizes at the nucleolus during vegetative development. In line #236, where 45S rDNA signal is strongly reduced, all signals remain exclusively located at the nucleolus (Scale bar: 5 μm).

The Col-0 accession harbors four allelic variants of 45S rDNA which are associated with either NOR2 (VAR1 and 3) or NOR4 (VAR2, VAR3, and VAR4) (Figure�2C and Pontvianne et�al., 2010; Chandrasekhara et�al., 2016). Investigation of genomic abundance of the 45S rDNA variants (Figure�2B) revealed that our mutagenesis approach caused a range of gene dosage variation of the 45S rDNA repeats across each independent line. Further, we investigated via reverse transcriptase polymerase chain reaction (RT-PCR)�whether the expression levels of the different 45S rDNA variants were altered and found qualitative changes in variant expression in the latest generation analyzed (T7). For instance, we observed a strong expression signal of VAR4, the least abundant variant, in seedlings of line #236, while VAR1 appears more actively transcribed in rosettes of both LCN lines.

From the T1 generation, we selected two lines with particularly low CN, lines #236 and #289 (Figure�2A, henceforth termed as LCN lines), and allowed these lines to self-fertilize for six generations, after which we recovered plants with CN variation ranging from 7% to 17% (line #289) and 11%–21% (line #236) of WT (Figure�2B). In the LCN lines (#236 and #289 T7 generations), effects on plant development were characterized from germination onwards (Supplemental Figure S1), where we found that the majority of seeds established a healthy seedling (∼80%) and a similar percentage of seedlings successfully underwent their complete life cycle. In this study, we have focused on the WT-like plants to improve understanding of how a multicellular organism, with numerous cell-fate transitions throughout its lifecycle, can successfully complete its lifecycle with only ∼10% of its 45S ribosomal RNA genes, under the growth conditions used.

To further confirm the reduction of 45S rDNA copies in line #236, we performed fluorescence *in situ* hybridization (FISH) probing the 45S rDNA repeats in line #236 at the T7 generation. As previously shown, we found that both NOR2 and NOR4 are actively transcribed in young seedlings of *A. thaliana* Col-0, where one or two large signals localize at the nucleolus, indicating that both NORs are available for transcription (*n* = 10 plants). However, during the course of WT development, NOR2 becomes progressively silenced (Chandrasekhara et�al., 2016) and moves further away from the nucleolus, eventually organizing in form of chromocenters at the nuclear periphery. NOR4 remains in proximity to the nucleolus, typically localized at two large chromocenters on the sides of the nucleolus (*n* = 12).

We observed a strong reduction in signal intensity (Figure�2D, *n* = 50), confirming the drastic loss of 45S rDNA copies in line #236. NOR signals are localized at the nucleolus, although the fluorescence appears more diffuse within the nucleolus itself, indicating loss of the highly condensed chromatin organization of the NORs. During vegetative development of line #236, the 45S signals still localize inside the nucleolus, indicating that copies from both NORs appear to remain available for transcription, similar to what is observed in the *fas1 fas2* mutant (Pontvianne et�al., 2013). Although some chromatin condensation is still visible (i.e. the rounder signals within the nucleolus), this localization of NOR2 within the nucleolus suggests that the 45S rRNA gene copies of NOR2 may remain available for transcription in line #236 (*n* = 30) as a potential mechanism of gene dosage compensation.

### While chromatin organization is strongly altered, rRNA homeostasis remains unchanged

We investigated whether transcription of rRNA or its accumulation is altered in the LCN plants, particularly during seedling development, when more copies of rRNA genes are actively transcribed. Hence, we quantified rRNA levels in line #236 by two methods. We performed an RNA gel blot using probes targeting either the functional rRNA (18S) or the precursor 45S (Figure�3B). Our analysis shows that apart from minor differences in the 5′-region of the transcript, there is no major alteration in rRNA level accumulation between the LCN lines and the WT control. We also performed an absolute quantification of rRNA molecules (using RNA spikes, see Materials and methods Section, Figure�3C) on pooled seedlings of line #236 in a previous generation (T4), and found no difference in rRNA accumulation with WT. Cell size was also measured to exclude any underlying difference in global rRNA accumulation: leaf protoplasts were isolated and cell size measured. A 6% decrease in cell size was found in line #236 (Supplemental Figure S1), which is likely negligible when comparing global RNA accumulation per biomass.

**Figure 3:**
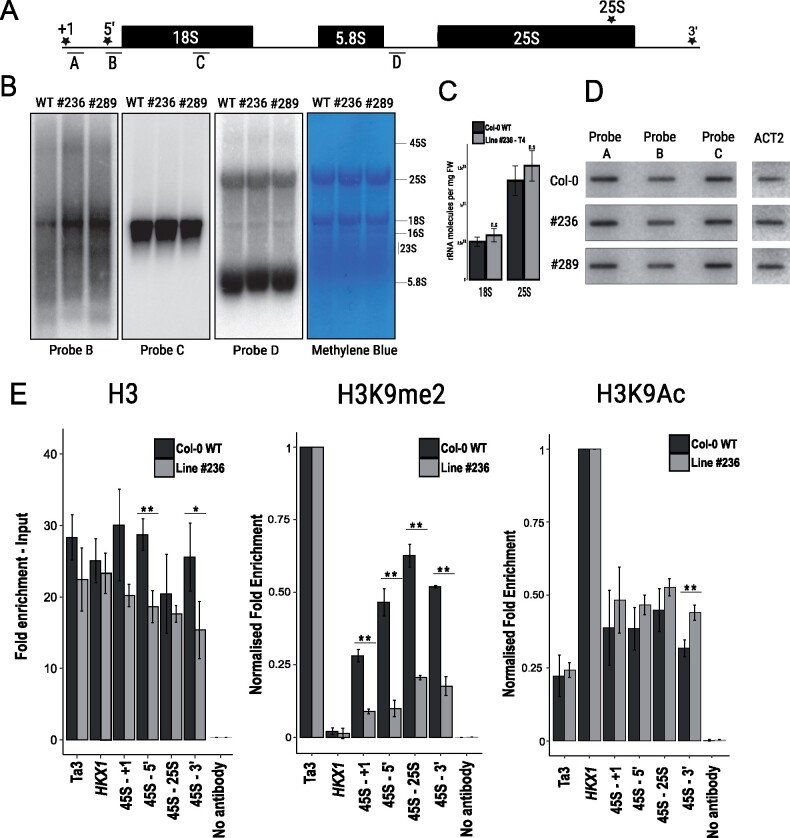
Synthesis of 45S rRNA appears largely regulated by chromatin organization. A, 45S rDNA locus. Letters indicate the probes used for RNA gel blots (B) and transcription run-on (C). Stars represent regions amplified by ChIP-qPCR. B, RNA gel blot analysis shows accumulation of 45S and other ribosomal RNAs in WT Col-0 and the LCN lines #236 and #289, letters indicate probes used as shown in (A), Methylene Blue is shown as loading control. Worth noticing that the plastid 16S and 23S rRNAs result equally represented in the LCN mutants and show a WT-like stoichiometric ratio with the 45S-derived rRNAs. C, Absolute quantification of 18S and 25S rRNA molecules in WT and #236 (T4 generation). No difference in the accumulation of either was detected (Student *t* test, bars represent standard error among biological replicates, *n* = 3 biological replicates). D, Nuclear transcription run-on assay shows transcription rate for 45S rRNA in WT Col-0 and the LCN lines #236 and #289. *ACT2* transcript was used as control. E, ChIP-qPCR shows differential enrichment of global H3, H3K9me2 (silencing mark) and H3K9Ac (active mark) across the 45S loci. Ta3 and *HXK1* were used as controls for a silent retrotransposon and a transcriptionally active gene, respectively. H3 occupancy (left) was determined relative to input. Fold enrichments for H3K9me2 (middle) were normalized against heterochromatic control Ta3; fold enrichments for H3K9Ac (right) were normalized against euchromatic control HXK1. (Student *t* test, bars indicate standard error among biological replicates, **P < 0.01, *P < 0.05, *n* = 3 biological replicates, no antibody control = average of 45S no antibody amplicons).

Remarkably, this suggests that drastic reduction of 45S CN does not affect levels of rRNA accumulation. Hence, we hypothesized the presence of a gene dosage compensation mechanism affecting transcription which allows rRNA accumulation in the LCN lines to be similar to WT, despite a loss of >90% of 45S rRNA genes. We considered whether such a gene dosage compensation mechanism might be acting through higher rates of transcription at active repeats, and/or by chromatin reorganization allowing additional rDNA repeats to be activated.

To investigate the possibility of a gene dosage compensation mechanism involving increased transcription rates, we performed nuclear run-on transcription assays on line #236 (T7), and WT: we probed the 5′-region of the transcript, which appears more abundant in #236 than in WT from our RNA gel blot analysis, but found no evidence of an increase in the global rate of transcription of rRNA in the CN depleted line (Figure�3D). Taken in combination with the steady state rRNA levels remaining the same, this indicates that rRNA stability remains similar in the CN depleted lines. However, it cannot be excluded that rate of transcription per activated 45S rRNA gene copy could potentially be increased, i.e. through increased Pol I loading or occupancy (French et�al., 2003; Lawrence et�al., 2004).

We further investigated whether chromatin organization changes were occurring in this same line #236, which could allow for rRNA accumulation to be as high as WT. We used chromatin immunoprecipitation (ChIP) against active (H3K9ac) and silencing (H3K9me2) marks which are known to be present on the rDNA repeats, as well as global H3 as a proxy for nucleosomal occupancy on the repeats (Benoit et�al., 2019). qPCR was then used to estimate the enrichment of both marks across the 45S rRNA gene, relative to a euchromatic control (*HXK1*) and a heterochromatic control (Ta3––LTR retrotransposon). We demonstrate that the enrichment of H3K9me2 was significantly reduced at the 45S rDNA loci in the LCN lines, while a trend of higher enrichment of H3K9Ac was also observed at the 3′-end of the repeats (Figure�3E). Furthermore, the dramatic loss of H3 occupancy at the 5′-region of the 45S rRNA gene suggests a more relaxed chromatin state of the regulatory regions which is consistent with active rDNA repeats devoid of nucleosomes. Taken together, our data indicate that gene dosage compensation of rRNA levels occurs despite rDNA CN depletion. We suggest that this is most likely due to increased frequency of transcriptional activation of rDNA copies in LCN lines, occurring predominantly through a relaxation of chromatin organization and loss of silencing marks.

### Long-read sequencing of two independent LCN lines indicates that genome architecture is largely preserved

To investigate the chromosomal integrity of the genome in our LCN lines, we performed long-read Nanopore sequencing (Table�1) to detect possible deletions, insertions or duplications in the CN depleted lines #236 (T6) and #289 (T5) (Figure�4). Analysis of the coverage of the nanopore reads, of the CN depleted lines versus WT, identified five loci with a coverage fold change <0.5 or >1.5 (Figure�4A). The two loci with loss of coverage in the CN depleted lines relative to WT mapped to the two 45S rDNA repeats loci annotated in TAIR10 (NOR4 is not annotated in TAIR10, with the 45S genes located in Chr2, and Chr3 in TAIR10 reference genome), confirming the loss of 45S rRNA genes in those two lines (∼20% coverage in both lines versus WT, Figure�4D), consistent with our qPCR and FISH assays (Figure�2).

**Figure 4:**
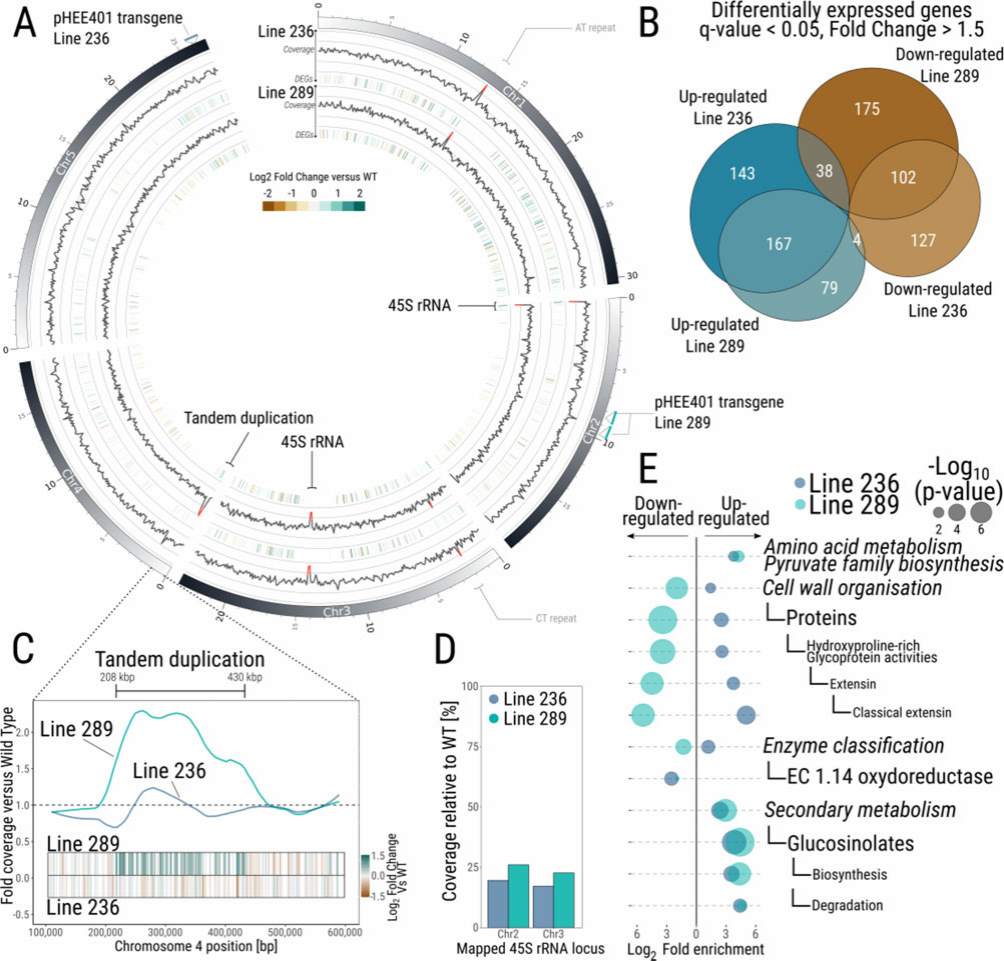
Genome integrity and transcriptome effects of 45S contraction. A, DNA coverage and gene expression analysis of two independent LCN lines compared with WT. For both LCN lines, outer layer represents fold change of the DNA coverage compared with WT, inner layer the identified DEGs (q-value <0.05, fold change >1.5). Coverage >1.5 or <0.5 versus WT, representing duplications and deletions, respectively, are highlighted in red. B, Euler diagram representing the number of DEGs between the two LCN lines and WT. C, Close-up of the duplicated region identified in line 289. *Y*-axis represent the fold coverage versus WT, *X*-axis represents the position on Chromosome 4. Bars indicate genes, color coded based on their expression level versus WT. D, Coverage of 45S rDNA gene loci in LCN lines relative to WT assessed by Nanopore sequencing. E, MapMan enrichment analysis of bins significantly enriched for up or downregulated genes in both independent LCN lines. *X*-axis represent the fold enrichment of each significant bin, left: downregulated genes, right: upregulated genes, the size of the circles corresponds to the −log_10_ adjusted *P*-value.

**Table 1:** Summary statistics of the nanopore sequencing runs

Sample	Number of reads	Mean read length (bp)	Mapped %	Mean coverage	Mean mapping quality
Line 236	1,043,643.00	3,868	98.01	30.17	55.95
Line 289	1,237,074.00	4,097	96.74	37.73	56.19
WT	569,275.00	6,570	92.21	28.97	54.52

Of the three other loci displaying differences, two mapped to repetitive elements on Chromosomes 1 and 3 (92 bp AT repeat in Chromosome 1 starting at position 13,085,467 and a 70 bp CT repeat in Chromosome 3, starting at position 2,258,906). We consider that these likely represent background differences with the Col-0 WT line used as control for coverage analysis, which was not the original line used for pHEE401 transformation. A single, large duplication event (∼220 kb, at positions 208,000–430,000 and containing 51 genes) was found in line #289 on chromosome 4 (Figure�4C), indicating that, at least for one of the two LCN lines, genome integrity has been affected. To ascertain that this duplication is correlated with loss of 45S rDNA, we designed primers spanning the duplication segment, which allowed us to confirm its appearance at the T5 generation (Supplemental Figure S2). This suggests that genome integrity has possibly been affected by loss of 45s rDNA, as described in another line which features loss of 45S rDNA (Picart-Picolo et�al., 2020).

### Genome-wide gene expression changes following 45S rDNA CN reduction

While the impact of a major reduction of 45S CN on genome architecture appears limited, we further analyzed the transcriptome of 7-day-old seedlings (with three biological replicates) of the two LCN lines. This revealed the presence of transcriptome differences between WT seedlings and the two LCN lines (Figure�4, A and B). Indeed, the seedlings of the LCN lines #236 and #289 had 581 (233 down- and 348 upregulated) and 565 (315 down- and 250 upregulated) differentially expressed genes (DEGs, q-value <0.05 and fold change >1.5), respectively, when compared with WT. About 50% of the DEGs were detected in both LCN lines (Figure�4B, Supplemental Data Set 1). Similarly, MapMan4 bin enrichment analysis identified some consistent responses between the two LCN lines (Usadel et�al., 2009; Schwacke et�al., 2019). We identified 12 bins enriched for DEGs in both LCN lines (Figure�4E;Supplemental Data Set 1), representing 54% of enriched bins in both lines #236 (22 bins) and #289 (22 bins), respectively.

Among those enriched bins were functional processes and enzyme pathways such as cell wall organization, oxidoreductases (including the Cytochrome P450 family), biosynthesis and degradation of glucosinolates, and biosynthesis of pyruvate family amino acids. The enrichment for DEGs in these bins suggests a possible dysregulation of key pathways, such as glucosinolate metabolism or cell wall formation in the LCN lines. Genes involved in cell wall organization displayed opposite expression profiles between the two independent LCN lines, with an up- and downregulation of genes involved in this process in line #236 and #289, respectively.

Finally, 35% of the genes located within the novel tandem duplication detected on Chromosome 4 of line #289 were found to be upregulated (18 out of 51 genes, mean fold change of the 51 genes within locus = 1.5; Figure�4C) displaying a positive gene-dosage response. Conversely, only two genes in this locus were dysregulated in line #236 (2 downregulated genes out of 51). The mean fold change was 0.93 in line #236, which did not contain the tandem duplication (Figure�4C).

## Discussion

### Severely depleted rDNA CN is compatible with plant viability

We hypothesized that targeting the tandemly arrayed 45S rDNA repeats with Cas9 endonuclease in the female gametophyte would cause DSBs across the NORs, causing a transgenerational reduction of 45S rRNA genes. Supporting this we found a high degree of CNV in the transformant T1 population, indicating that Cas9 activity had caused both loss and gain of 45S rDNA copies as hypothesized. The Col-0 accession harbors approximately 375 copies per NOR. In our study, we aimed to reduce 45S CN by allowing Cas9 activity in the EC by maintaining these generations of these transformed lines until a reduction in CN was no longer detected. This was achieved at the T7 generation in lines #236 and #289, where rDNA CN plateaued at ∼10%. By using transgenerational Cas9 activity, we have demonstrated that, by the eighth generation, about 80% of plants in both #236 and #289 can fully undergo their lifecycle despite having only ∼10% of rDNA copies compared to WT (i.e. ∼25/30 copies per NOR). This finding allows us to definitively conclude that as little as 50 rDNA copies (10% of WT) per haploid genomes are sufficient for viability in *A. thaliana* accession Col-0, which was previously proposed to be 15%–20% (Pontvianne et�al., 2013) but impossible until now to causatively establish.

It remains to be determined whether the lowest functional CN is absolute or relative to the WT CN. Since *A. thaliana* accessions display large CNV, spanning from approximately 500 copies to approximately 2,500 (Rabanal et�al., 2017), generating LCN lines in accessions with higher or lower 45S CN will help to address this question. In *A. thaliana*, such a drastic loss of rDNA copies has only been observed in the *fas1 fas2* mutant (Mozgova et�al., 2010), which showed a 45S rDNA CN reduction of ∼80% of WT. However, the question of minimum 45S CN compatible with viability was confounded by the pleiotropic phenotypes exhibited by the *fas1 fas2* mutant, such as loss of telomeres on all chromosomes (Mozgova et�al., 2010). Our analysis of the 45S rDNA variants––which are associated with either NOR2 (VAR1 and VAR3) or NOR4 (VAR2, VAR3, and VAR4) (Chandrasekhara et�al., 2016; Mohannath et�al., 2016) suggests that loss of CN from either NOR occurred randomly in each independent line, as one could expect due to the sequence identity, and repetitive nature of the 45S rDNA repeats.

Although we demonstrate here that plants with as little as 50 rDNA copies per haploid genome are able to complete their lifecycle, the presence of ∼20% of unviable seedlings within the progeny of the two independent low CN lines remains to be mechanistically characterized (Supplemental Figure S1). Further, we located the insertion site(s) of the transgenic Cas9 cassette in both lines using the nanopore data. We found homozygous insertions (two insertions in #289, mapping both to chromosome 2, position 9.73 Mb and 8.58 Mb, and one insertion in #236, mapping to chromosome 5, position 26.04 Mb, Figure�4A). In line #289, the first transgene insertion is located within the promoter of *AT2G22860*, and the second insertion in the 5′-UTR of *AT2G19880* but neither are associated with expression changes. In line #236, the insertion is located in an intergenic region between genes *AT5G65165* and *AT5G65170*, none of which are differentially expressed. Hence, we consider that the insertion sites of the transgenic cassette are unlikely to affect the two low CN plant phenotypes and/or viability, and that the 20% of unviable seedlings found in both LCN lines is likely due to other mechanisms.

### Altered chromatin organization of 45S rDNA ensures gene dosage compensation of rRNA transcription levels

One of the key questions we investigated was whether reduction in 45S rDNA CN causes alteration to rRNA transcription levels, particularly during critical stages of development (e.g. seedling establishment). However, no difference in rRNA levels was found in either of the two LCN lines both for mature rRNA accumulation, as well as the precursor rRNA transcript. Although major cell size differences could lead to erroneous absolute quantifications based on biomass, we only found a small (∼6%) difference in cell size in line #236 versus WT (Supplemental Figure S1). This indicates that biomass-based quantification of rRNA transcript abundance is appropriate in this system; however, this finding also presents interesting new avenues to be explored in the future detailed phenotypic characterization of LCN lines.

Following the analysis of steady-state levels, nuclear run-on assays were used to measure *in vitro* transcription rates of rRNA. This also indicated little difference in rDNA transcription rates between WT and LCN lines. To identify the underlying mechanism for gene dosage compensation of rRNA levels in the 45S rDNA CN depleted lines, we probed the epigenetic landscape of the rDNA locus and found statistically significant changes in the chromatin organization of the repeats. Our results suggest that the loss of enrichment in silencing marks such as H3K9me2 may be sufficient to allow for gene dosage compensation of rRNA transcription to occur in the LCN lines resulting in similar rRNA levels as in WT plants, despite the dramatic loss of 45S rRNA CN. It is plausible that removal of silencing marks at rRNA allows for WT-like transcription levels of rRNA, without the apparent necessity of altering transcriptional rates. Despite the large reduction in rDNA copies, such increased levels of rRNA levels may arise through the action of enhanced histone demethylase activity at rDNA loci (Pontvianne et�al., 2012). This hypothesis remains to be tested by investigating the levels of activity of different histone lysine methyltransferases and histone demethylases which could be involved, as well as probing global enrichments of heterochromatin in comparison to WT.

Taken together, our findings support a model for gene dosage compensation of rRNA transcript levels in *A. thaliana* through chromatin organization, where the transcriptional activity of Pol I itself is controlled largely through epigenetic modification, as previously suggested (Grummt and Pikaard, 2003). It cannot be excluded, however, that increased loading of Pol I on the rDNA promoter could also aid in maintenance of transcription, as reported in yeast (French et�al., 2003).

The relative proportion of active/inactive and silent rDNA genes is still unknown: the FISH images we collected suggest that a degree of chromatin condensation is still present (i.e. from the larger round signals inside the nucleolus). However, chromocenters appear hollower and less condensed than in WT nuclei, similarly to what was observed in the *atxr5 atxr6* mutants, which suffer a conspicuous loss of heterochromatin (Feng et�al., 2017).

### Chromosome-segment duplication: a symptom of genome instability or a mechanism to preserve genome integrity?

We considered that Cas9 activity acting on the rDNA repeats in the female gametophyte, coupled with transgenerational loss of rDNA copies, could induce and aggravate genome instability through extensive DNA damage, and loss of large amounts of genomic DNA. We used long-read Nanopore sequencing to determine any major chromosomal rearrangements, deletion or insertions. Other than a ∼220-kb segmental duplication on chromosome 4 of line #289, we found no major signs of genomic changes. However, one of the most upregulated genes in both lines is *AT3G61010* (log2 fold change >7). This gene is part of a family of ferritin/ribonucleotide reductases genes that have been linked to genotoxic stress in Arabidopsis (Roa et�al., 2009). The upregulation of that gene could be a direct result of potential genotoxic stress induced by either the expression of the Cas9 transgene in the EC or by the loss of rDNA copies itself. Picart-Picolo et�al., 2020 have reported that Line 6 from a *fas1 fas2* population (Pavlištov� et�al., 2016), which bears 20% of rDNA genes, harbors a chromosome segment duplication observed at the heterochromatic knob region of chromosome 4 (Picart-Picolo et�al., 2020). Curiously, the duplication we retrieved in line #289 appears in a neighboring region of the heterochromatic knob on the same chromosome. On the other hand, the duplication in Line 6 is accompanied by a large number of smaller tandem duplications across the genome, which was not found in our LCN lines.

This leaves open the question of further determination of the level of genomic instability in lines #236 and #289. However, these segmental duplications occurred in neighboring regions of two independent lines (#289 and Line 6), obtained in two completely different approaches. This suggests the possible presence of a fragile region (e.g. with rDNA CN dependent fragile sites) which might be crucial for genomic structural integrity and which becomes duplicated following large loss of repetitive DNA (or rDNA) content. The heterochromatic knob on chromosome 4 *hk4s* is the only region of the *A. thaliana* genome which features a Topologically Associated Domain-like structure (Grob et�al., 2013; Pontvianne and Grob, 2020), suggesting that this region could play an important role in the structural dynamics of the genome of *A. thaliana*. Furthermore, our results suggest that the genome appears well-buffered against loss of rDNA copies; however, DNA damage and repair pathways in these lines remain to be investigated, together with global heterochromatin enrichment, to provide a full view of the landscape of genome stability in these lines.

We consider that these novel rDNA depleted lines provide exciting new tools to (1) investigate the extent of genomic instability singularly caused by loss of 45S rDNA CNs, (2) to characterize the effects of extensive DNA damage on rDNA loci in the female gametophyte, (3) to elucidate the role of 45S rDNA in plant cellular senescence, and (4) to investigate the dynamics of rDNA CNV when backcrossing to WT and/or with other accessions with reduced 45S CN.

### The transcriptome of LCN lines shows significant effects of 45S rDNA CN depletion

The transcriptome analysis of both independent LCN lines revealed approximately 570 dysregulated protein-coding genes compared with WT with a fold change >1.5 (Figure�4D). Such transcriptome difference could be caused by the loss of rDNA copies itself or by chromatin remodeling due to nucleolus reorganization, possibly to facilitate the activation of all remaining 45S rDNA copies. In this context, we hypothesized that the epigenetic machinery may be responsible for maintaining ribosomal RNA homeostasis (Figure�3), through aberrant deposition/maintenance of epigenetic marks throughout the rest of the genome. Genome-wide ChIP on histones and histone marks can be used to better characterize the transcriptome effects observed due to rDNA depletion. Genome-wide aberrant epigenetic marks could lead to changes in gene expression between independent lines. Furthermore, as shown in Figure�4B and E, 311 of the differentially expressed genes are shared between the 2 independent LCN lines, leading to 12 enriched common pathways (Figure�4E). Those results indicate that 45S rDNA contraction likely leads to transcriptome changes, with a common set of rDNA CN-sensitive genes and pathways. Of note also is the dysregulation of glucosinolate metabolism in both lines, suggesting elevated cellular stress which might be connected to pathogen response pathways, as observed in Line 6. However, the precise cause and effect of the dysregulation of glucosinolate biosynthesis and degradation genes remains to be characterized. Recently, glucosinolate re-mobilization following Carbon starvation was observed in Arabidopsis (Brandt et�al., 2018), indicating that the glucosinolate metabolism is likely to play a role in adaptation to abiotic stress, in addition to its well-known role in defense.

Picart-Picolo et�al., 2020 highlighted that Line 6 displays approximately 350 DEGs (Picart-Picolo et�al., 2020). The DEGs appear to be correlated with the occurrence of duplication events. Our study supports this hypothesis, since the single duplication event found in line #289 indeed correlates with higher expression levels of 35% of the genes within the duplicated locus (18/51 genes upregulated, Figure�4C, mean fold change of genes within the region of 1.5 versus WT). However, this cannot explain the deregulation of the remaining approximately 550 deregulated genes, or indeed of the 581 DEGs in line #236 which does not contain duplications despite a similarly low 45S CN. Conversely, only 16 DEGs are common between line 6 and our low CN lines #236 and #289. Hence, our analysis reveals an effect on the transcriptome arising simply from rDNA CN depletion, similar to effects observed in *Drosophila* (Paredes et�al., 2011). The mechanistic basis for such transcriptome deregulation remains to be further investigated in future studies.

In this study, we present an approach and tools to elucidate the precise role of 45S rDNA CN in plants. Indeed, our approach represents a simple, “clean” method to modify rDNA CN without off-target modifications. A single transformation event allowed us to generate a population of plants with rDNA CN ranging from ∼20% to 160% of those of WT. Intriguingly, we present evidence that gene dosage compensation of rRNA levels is tightly regulated, most likely by chromatin remodeling, with a similar rRNA accumulation in LCN lines despite the loss of hundreds of 45S copies. The possible impacts of 45S CN depletion on protein dynamics, genome integrity, plant reproduction, development, and fitness are fertile avenues for further investigation using the novel 45S LCN lines (Figure�5).

**Figure 5:**
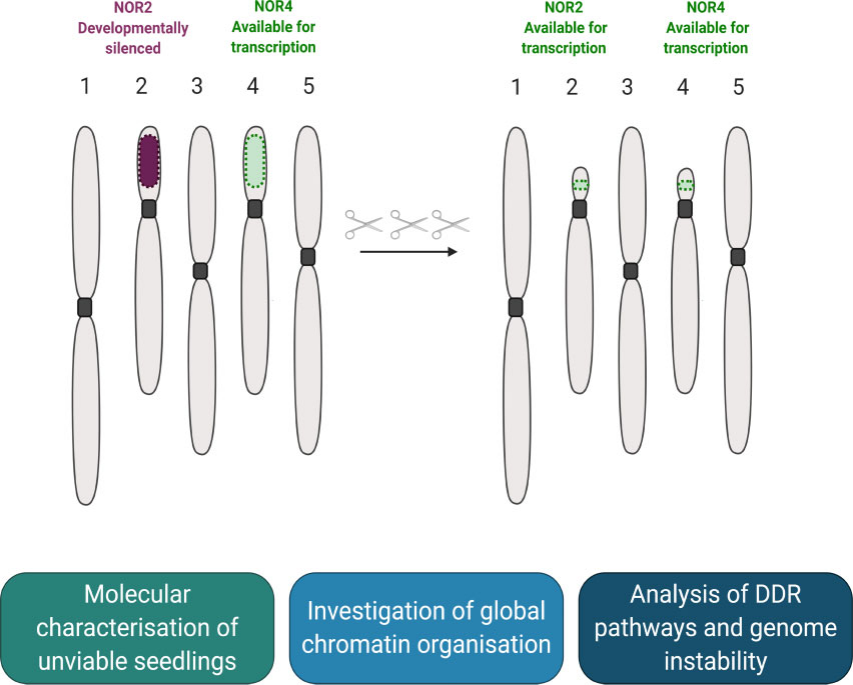
Effects of rDNA CN reduction on rRNA transcription. Analysis of rRNA transcription in our lines revealed that gene dosage may be maintained by rendering both NORs competent for transcription potentially through the removal of silencing histone marks. The rDNA LCN lines generated can be further used to advance understanding of the functional role of rRNA genes in cellular and developmental processes.

Gene dosage compensation systems have arisen in different evolutionary contexts to adjust transcript levels in response to changes in gene dosage (e.g. as arises on sex chromosomes) (Veitia et�al., 2013). Our results reveal a gene dosage compensation mechanism in Arabidopsis for rRNA transcript levels when rDNA CN is altered, which differs from classical genetic studies in maize where changing the dosage of NORs was found to have a dosage effect on NOR RNA levels (Lin, 1955). Conversely, Buescher et�al., 1984 found that a maize line with two NORs did not affect the levels of rRNA, which is consistent with our findings of gene dosage compensation effects on rRNA levels in *A. thaliana.*

Finally, our study adds to the growing body of knowledge on use of CRISPR-Cas for study of plant functional genomics (Lynagh et�al., 2018; Beying et�al., 2020). Our novel approach paves the way for research on understanding roles of crop rDNA repeats, including in species where genomic architecture is more complex than Arabidopsis (e.g. with a higher abundance of repetitive elements, allopolyploidy, and high functional redundancy).

## Materials and methods

### Plant materials and growth conditions

*Arabidopsis thaliana* accession Col-0 was used for all experiments. Unless otherwise specified, plants were grown on either 1% w/v Murashige and Skoog medium (seedling samples) or sown onto a mixture of soil, perlite, vermiculite (5:1:1) (leaves samples). Long day conditions (16-h light at 21�C and 8-h dark at 18�C) were used with white LED lights with Photosynthetically active radiation of ∼100 �mol.m^−2^.s^−1^ photons.

### Generation of T1 Cas9 transgenic plants

The vector used in this study (pHEE401) has been previously described in (Wang et�al., 2015). 18S-specific single gRNA was designed using CRISPR-P (http://cbi.hzau.edu.cn/crispr/) and cloned using the following oligonucleotides: 18S Fwd ATTGATACGCTCCTGGTCTTAAT and 18S Rev AAACATTAAGACCAGGAGCGTAT. Annealed oligos were directly inserted into pHEE401 using BsaI cut-ligation. Recombinant plasmids were electroporated into *Agrobacterium tumefasciens*, and Col-0 plants transformed using the floral dip method (Clough and Bent, 1998). Transformed seeds were surface-sterilized using chlorine gas and sown onto 0.5% Murashige & Skoog medium with 20 �g/mL of Hygromycin B. 

### DNA isolation and CN quantification

For genomic DNA extractions, single seedlings or 10 mm^2^ piece of rosette leaves were flash-frozen in liquid nitrogen and subsequently ground into a fine powder using a QIAGEN TissueLyser. Pulverized tissue was incubated at 65�C for 1 h in 2X CTAB extraction buffer (2% Cetyl-trimethyl-ammonium bromide, 1.4 M NaCl, 100 mM Tris–HCl pH 8.0, 20 mM Ethylenediaminetetraacetic acid (EDTA), 5 mM β-mercaptoethanol). Samples were incubated on ice for 2 min followed by addition of 1 volume of chloroform. Samples were vortexed thoroughly and centrifuged, supernatant was collected, and DNA was precipitated with ice-cold isopropanol. Samples were centrifuged and pellet washed with 70% Ethanol and air dried. DNA was suspended in 50 �L of sterile ddH_2_O. To quantify the number of 45S copies in the genome of WT and LCN lines, we followed the method described in (Rabanal et�al., 2017), using quantitative real-time PCR (Bio-Rad CFX™ thermocycler) in 5-�L reaction volume with SensiFast Sybr No-ROX kit (Bioline). CN per haploid genome was calculated by comparing the threshold cycle from the 18S subunit of the 45S repeats with the threshold cycle from the single copy genes *TZF1* and *HXK1* using the following formula

$$\mathrm{CopyNumberperhaploidgenome}=\frac{2^{Ct\mathrm{Singlecopygene}}}{2^{Ct18S}}$$

CN per haploid genome was normalized on WT values to express the CN in our various lines as a percentage of WT CN.

### Absolute quantification of ribosomal RNA molecules

For the quantification of rRNA molecules, we used the protocol described in (Piques et�al., 2009; Pal et�al., 2013; Ishihara et�al., 2017; Vaid et�al., 2020) with modifications. Seven-day-old seedlings of WT and #236 lines were harvested and immediately flash-frozen in liquid nitrogen, with three biological replicates. The frozen biomass was ground into a fine powder using a ball mill (QIAGEN TissueLyser II). Aliquots of ∼20 mg of ground frozen tissue were resuspended in 1 mL of Bioline ISOLATE II lysis buffer, and the suspension diluted 10-fold in lysis buffer. About 10 �L of that final dilution was used for RNA extraction. An RNA spike mix (Ambion ArrayControl spikes 2, 5, 6, 7, and 8) containing a known number of RNA molecules in the dynamic range of 3.9 � 10^7^ to 7.13 � 10^8^ per �L was added to the tissue prior to RNA extraction. RNA extraction was performed using Bioline ISOLATE II RNA kit following manufacturer’s instruction. RNAs were subsequently treated with DNAse1 (Sigma-Aldrich), and cDNA synthesis performed on 15-�L RNAs using Bioline SensiFast cDNA synthesis kit with random hexamers. The cDNAs were diluted 50-fold with sterile miliQ water before RT-qPCRs. qPCRs were performed in 5 �L reactions containing 1 �L of cDNA and 4 �L of SensiFast Sybr No-ROX kit (Bioline). Threshold (Ct) values obtained from the qPCR reactions against each RNA spike were used to generate a standard curve for each sample (*R*^2^ >0.96 for all reactions). Ct values from the 18S and 25S qPCR reactions were compared with the slope and intercept to obtain the absolute amount of RNA molecules for both rRNA transcripts. Finally, the number of rRNA molecules was normalized by the amount of biomass in the aliquots used for RNA extraction (Ishihara et�al., 2017). Since rRNA quantity could be affected by a reduction in rDNA CN. The spikes were added prior to RNA extraction and the results normalized on a biomass basis. Adding the spikes to a fixed amount of RNA rather than biomass could give erroneous results in the case of a differential expression of ribosomal RNAs. Indeed, since rRNAs represent the majority of total RNAs within a cell, normalizing by the amount of total RNA (as is done in RNA gel blots) could potentially erase the possible intrinsic difference in rRNA levels between samples, leading to the risk of false negative results.

### 3′-EST variant assays

gDNA from individual rosette leaves of T4 and T7 generations was extracted by CTAB as previously described, from lines #236 and #289. PCR was carried out as follows using primers P3–P4 (Supplemental Table S1) and MyTaq Red Mix by Bioline^�^: 94� for 5′, 25 cycles of 94� for 30″, 58� for 30″, and 72� for 60″.

For cDNA analysis of variants, RNA extracted as previously described, and treated with DNAse I by Invitrogen^�^ as per manufacturer’s instruction. Of about 1.5-�g RNA were retrotranscribed with SuperScript™ III First-Strand Synthesis System by Invitrogen^�^, following manufacturers instruction, PCR for variant analysis was carried out as follows using P3–P4 (Supplemental Table S1), and MyTaq Red Mix by Bioline^�^: 94� for 5′, 29 cycles of 94� for 30″, 58� for 30″, and 72� for 60″.

*ACT2* was amplified from cDNA using primers listed in Supplemental Table S1 and PCR was carried out as follows: 94� for 5′, 26 cycles of 94� for 30″, 58� for 30″, 72�.

### RNA gel blot and nuclear run-on assay

For RNA gel blot studies, total RNA was isolated from 7‐day‐old (after germination) pooled Col‐0 and LCN seedlings. RNA gel blot analyses were performed using 4 μg of total RNA for each sample. ^32^P‐labeled DNA probes were generated using primers listed in Table�1. For Run‐on transcription assays, nuclei were extracted from 1.2 g of 7‐day‐old pooled seedlings and isolated according to (Folta and Kaufman, 2007). The transcription reaction was carried out for 30 min at 25�C in 100-�L transcription buffer [60 mm 4-(2-hydroxyethyl)-1-piperazineethanesulfonic acid (HEPES)‐KOH pH 8.0, 60 mm KOAc, 10 mm MgCl_2_, 10 mm dithiothreitol, 20 U RNase inhibitor (Thermo Fisher Scientific, Waltham, MA, USA), proteinase inhibitor cocktail (cOmplete™, Roche) 150 �m ATP, CTP, GTP, 15 �m UTP, and 5 �L [^32^P]‐UTP (3000 �Ci/mmol)], as described in Tadini et�al. (2019). Of about 1 �g of DNA probes (A′, A, and B; Figure�2A) were blotted onto a Hybond‐N^+^ membrane (Amersham, Little Chalfont, UK) and hybridized with ^32^P-labeled RNA. DNA probes were generated using primers as listed in Supplemental Table S1.

### Nanopore sequencing and data analysis

Genomic DNA preparation was performed as previously described. DNA was further purified using Genomic DNA Clean & Concentrator kit (Zymo Research, USA). Qubit (dsDNA High Sensitivity (Thermo Fisher Scientific, USA) quantification was performed before library preparation using the 1D Genomic DNA by ligation kit SQK-LSK109 (Oxford Nanopore Technologies, UK), following manufacturer’s instructions. The R9.5 ONT flow-cell FLO-MIN106D (Oxford Nanopore Technologies, UK) was used with MinKNOW version 3.6.5, Guppy 3.2.10 through the software MinION release 19.12.5. Reads were aligned onto the Arabidopsis genome using minimap2 (Li, 2018). Next, the Arabidopsis TAIR 10 genome was split into 100 kb windows using bedtools make windows and the coverage counts of WT and lines #236 and #289 was calculated against the 100-kb windows using bedtools coverage (Quinlan and Hall, 2010) and normalized by the total number of reads. Normalized counts were analyzed using R, and the fold change per window versus WT calculated by dividing normalized counts of the LCN lines against WT. The final normalized fold change was calculated by dividing the fold change per window by the geometric mean of the fold change of all 100-kb windows for lines #236 and #289. The normalized fold change per window was visualized using Circos (Krzywinski et�al., 2009) and 100-kb windows with a fold change <0.5 or >1.5 highlighted as having differential coverage from TAIR10 genome.

### RNA extraction and transcriptome analysis

RNA extraction was performed on pooled seedlings (collected in triplicates at 7 days after germination) of Col-0 WT, #236, and #289 with WT phenotype grown on three Murashige and Skoog plates using Aurum™ Total RNA Mini Kit (Bio-Rad). RNA Integrity and purity were verified by gel electrophoresis and Nanodrop quantification, 10 �g of total RNA was concentrated and purified using RNA Clean & Concentrator-5™ (Zymo Research), and sent for sequencing to Novogene UK. Transcriptome sequencing was performed on mRNA-enriched RNA libraries using Illumina technology, 150-bp paired end reads, generating >21 million reads for each library. The reads were trimmed of residual adaptor sequences using Trimmomatic (Bolger et�al., 2014) and transcript abundance estimated using Kallisto (Bray et�al., 2016), using the latest reannotation of *A. thaliana* reference transcriptome (Araport 11). Differential expression was assessed by Likelihood Ratio Testing with Sleuth in R (Pimentel et�al., 2017) using “genotype” (i.e #236, #289, and WT) as factor in the full model, against a reduced model without genotype information. The minimum detection frequency filter was set to >0.3 to allow for detection of transcripts detected in at least one of the three genotypes. Transcripts were aggregated into genes during the Sleuth analysis. The comparison of the fits between full and reduced models for the abundance of each gene highlights those whose expression is more likely determined by the genotype than by the null hypothesis. Finally, log_2_ fold change approximations were extracted relative to WT using a Wald test, also in Sleuth. Genes with an absolute log_2_ fold change (b-value) >0.58, representing a fold change of >1.5 were selected and *P*-value correction performed using Benjamini–Hochberg on the *P*-values of the genes passing the fold change threshold. The fold change cutoff was selected since individual genotypes were grown on separate plates, potentially creating subtle batch effects. Hence, we only considered targets with a fold change greater than 1.5 as differentially expressed. In summary, genes with (1) a likelihood ratio q-value <0.05, (2) a Wald test q-value <0.05, and (3) an absolute log2 fold change (b-value) >0.58 were deemed significantly dysregulated. For MapMan analysis (Usadel et�al., 2009), the lists of up and downregulated genes, as well as the entire list of expressed genes (background) was used for bin enrichment. Genes were assigned into a MapMan bin structure using Mercator4 (Schwacke et�al., 2019). A MapMan bin was classified as enriched if the number of genes belonging to that bin in the up/down lists was statistically higher than expected from the background, using a two-tailed Fisher’s exact test and adjusted for multiple testing using Benjamini–Hochberg correction. Only bins enriched in both lines were retained for the analysis, to highlight possible similarities between the two independent LCN lines. The Log2 fold enrichment was calculated by dividing the number of observed versus expected genes in each bin.

### Fluorescence *in situ* hybridization

Nuclei were extracted from either pooled seedlings (5 days after sowing) or rosette leaves (20 days after germination) of WT and line #236. Tissues were fixed in cold 4% para-formaldehyde for 15 min at 4�C and washed twice in Tris Buffer (10 mM Tris–HCl pH 7.5, 10 mM EDTA, 100 mM NaCl). Nuclei were extracted by chopping tissues with a razor blade in Galbraith Buffer (Galbraith et al., 1983) and filtered through a 30-�M disposable filter (CellTrics, Sysmex, Germany). Filtrate was dripped onto a microscope slide and, after air-drying, slides were post-fixed in 2% formaldehyde in Phosphate Buffered Saline (PBS). Slides were then washed in 2� Saline Sodium Citrate (SSC) and treated with RNAse (1 h at 37�C) after which they were washed again in 2� SSC twice and further fixed in 2% formaldehyde. Following fixation, slides were washed with distilled H_2_O and dehydrated in ethanol series (70%, 90%, and 100%), air dried and hybridized with the appropriate probes. 45S rDNA probes were generated by amplification of 18S and 25S from Col-0 genomic DNA and cloned into pGEM-T Easy (Promega). One microgram of each probe was labeled with Cy3-conjugated deoxy Uridine TriPhosphates (dUTPs) (GE Healthcare) by Nick Translation (Roche) according to manufacturer instructions. Probes were resuspended at a concentration of 10 ng/�L in hybridization buffer (50% deionised formamide, 2� SSC, 10 mM sodium phosphate pH 7, 20% dextran sulfate), and further applied onto the slides which were denatured at 80�C for 2 min and hybridized overnight at 37�C. Post-hybridization washes were carried out at 60�C twice in 2� SSC, slides were mounted in Vectashield containing DAPI (Vector laboratories). Imaging was performed with a Zeiss Axioimager and captured with ORCA- Flash4.0 V2 Digital CMOS camera C11440 (HAMA-MATSU) maintaining the same exposure time and conditions.

### Chromatin immunoprecipitation

Seedlings of WT and line #236 were grown for 15 days after sowing and the areal parts including the meristem were pooled and collected in triplicates in ice-cold ddH_2_O. Chromatin crosslinking was performed in 1% formaldehyde under vacuum for 10′. Quenching was carried out with 0.1 M glycine and samples were placed under vacuum for further 5′ and washed twice with ice-cold ddH_2_O. Tissues were flash-frozen and ground in liquid N_2_ and ChIP was carried out as described in (Benoit et�al., 2019). Chromatin was precipitated using antibodies for H3 (Abcam, ab1791), H3K9me2 (Abcam, ab1220), and H3K9Ac (Actif Motif AB_2561017). qPCR analysis was performed with a Roche Lightcycler 480 in 10-�L reactions of Roche Lightcycler 480 SYBR Green I Master using either primers for controls (Ta3 and *HXK1*) or for different regions of the 45S locus. Primers are detailed in Supplemental Table S1. To adjust for different IP efficiencies between the independent ChIP experiments, IP levels were normalized to input for H3, and normalized to Ta3 for H3K9me2, and to *HXK1* for H3K9Ac.

### Cell size measurement

Leaf protoplasts were extracted from 10 leaves of 4-week-old rosette of *A. thaliana* plants in WT and #236 background (*n* = 5 from each genotype) using the “Tape-*Arabidopsis* Sandwich method” described in Wu et�al., 2009. For cell wall digestion, 1.25% of Cellulase Onozuka R-10 (Serva) and 0.3% of Macerozyme R-10 (Serva) were used. Protoplasts were washed with W5 solution [5 mM Glucose, 154 mM NaCl, 125 mM CaCl_2_, 5mM KCl, 5 mM MES (Sigma-Aldrich), and imaged using an Olympus BX61]. Thirty-seven and forty-five pictures containing approximately 10,000 protoplasts were analyzed using ImageJ (https://imagej.nih.gov/ij/) to calculate cell size. The images were segmented using the "find edges" process and a color threshold. Touching cells were separated from the segmented binary images using the watershed process. Cell size was then measured using the analyze particles option.

### Data availability

Sequencing files for the nanopore and RNA-sequencing reads are available in Sequence Read Archive (https://www.ncbi.nlm.nih.gov/sra) under BioProject ID PRJNA640267.

### Accession numbers

Gene models used in this article can be found in the Arabidopsis Genome Initiative database under the following accession numbers: *HXK1* (*AT4G29130*), 18S (*AT2G010101*), and *TZF1* (*AT2G25900*).

## Supplemental data

The following materials are available in the online version of this article.

Supplemental Figure S1. Plant viability in LCN lines.

Supplemental Figure S2. Identification of chromosome segment duplication in line #289.

Supplemental Table S1. Primers used in this study.

Supplemental Data Set 1. Differentially expressed genes in lines #236 and #289 compared to WT; Mapman4 enriched bin analysis for lines #236 and #289.

## Supplementary Material

koab020_Supplementary_Data

## Notes

A.F., F.L., and C.S. designed the research; A.F. and F.L. performed the research in laboratory of C.S. (funded by Irish Research Council and Science Foundation Ireland), with support from M.M.H., J.F., P.R., R.S., P.M.K., and G.B.; A.V.P. and F.P. hosted and supervised F.L. as visiting researcher for experiments on cytology/ChIP and Nanopore sequencing; L.T. performed RNA gel blots and nuclear run on experiments in lab of P.P.; F.L., A.F., and M.M.H. analyzed the data; F.L. drafted the paper with assistance from A.F. All authors provided comments on manuscript drafts; manuscript was finalized for submission by F.L. and C.S.

The author responsible for distribution of materials integral to the findings presented in this article in accordance with the policy described in the Instructions for Authors (https://academic.oup.com/plcell) is: Charles Spillane (charles.spillane@nuigalway.ie).
