# Supplementary material for: Gene dosage compensation of rRNA transcript levels in Arabidopsis thaliana lines with reduced ribosomal gene copy number
Source: Plant Cell. 2021 Feb 2;33(4):1135–50. doi: 10.1093/plcell/koab020 (PMC8225240; doi:10.1093/plcell/koab020)
Supplement: koab020_Supplementary_Data [file koab020_supplementary_data.zip › SuppTable1_Primers.pdf]

Primer Name	5' - 3' sequence	Experiment/assay
18s gRNA F	ATACGCTCCTGGTCTTAAT	guide RNA
18s gRNA R	ATTAAGACCAGGAGCGTAT	guide RNA
HXK1-F	aggagctcgtctctctgctg	Single Copy gene for CN normalisation
HXK1-R	GCTCAAACAATCCACCATCC	Single Copy gene for CN normalisation
18S Cas9 F	TGAAAGACGAACAACTGCGAAAG	CN quantification
18S Cas9 R	TGGTTGAGACTAGGACGGTATCT	CN quantification
TZF1_F	AGCTGAGAGCGGAGATGTATGC	Single Copy gene for CN normalisation
TZF1 R	TCCAAGTCGACTCAACCGAGTG	Single Copy gene for CN normalisation
+1-2745F :	TTTACCAGAAAATAGGATTTAGTATCCTTATG	ChIP (Earley et al, 2010)
+1-2830R :	CGGTGCCAAGAAAAAAGAAATACTT	ChIP (Earley et al, 2010)
5'ETS-4241F:	TTGGAACGATTGATGATTTTGAGT	ChIP (Earley et al, 2010)
5'ETS-4316R:	GCCTACGAACACTAGCTATCCGATC	ChIP (Earley et al, 2010)
25S-10062F:	TGTTCACCCACCAATAGGGAA	ChIP (Earley et al, 2010)
25S-10132R:	TCAGTAGGGTAAAACTAACCTGTCTCAC	ChIP (Earley et al, 2010)
IGS-573F:	GTCAAACACTTGGTGATATGAACACA	ChIP (Earley et al, 2010)
IGS-623R:	GCATGGGTTTGTCATATTGAACGT	ChIP (Earley et al, 2010)
3'ETS-84F:	GAATTCCCAACTTTACACGAGCTC	ChIP (Earley et al, 2010)
3'ETS-134R:	AAGTAATAACATTTAACCTCGAGAGACGAG	ChIP (Earley et al, 2010)
18s - SeqF	ACCCCGACTTATGGAAGGGA	FISH Probe
18s - SeqR	CCGAACACTTCACCGGATCA	FISH Probe
25S - SEQ_F	TGACTTGGGTATAGGGGCGA	FISH Probe
25S - SEQ_R	GGGAGGGTCGAATCTTAGCG	FISH Probe
25S_3'_P3	GACAGACTTGTCCAAAACGCCCACC	rDNA variants
25S_3'_P4	CTGGTCGAGGAATCCTGGACGATT	rDNA variants
Probe A'_F	TTGAGGGAGTCTGGGCAGTC	Northern Blots/Run On
Probe A'_R	CACCCAAACGGCCTAGGAGA	Northern Blots/Run On
Probe A_F	GCTTGTACGGCTTTGGCTCG	Northern Blots/Run On
Probe A_R	ACCGTCATCTTTTGCCCGAA	Northern Blots/Run On
Probe B_F	TCCCTTCGGTCGGCGA	Northern Blots/Run On
Probe B_R	CCTGGTAAGTTTCCCCGTGT	Northern Blots/Run On
Probe C_F	TGGCATTCGCCTCCCCG	Northern Blots/Run On
Probe C_R	GGGCTCTTCCCGGTTCG	Northern Blots/Run On
P3_Variants	GACAGACTTGTCCAAAACGCCCAC	3' ETS Variant
P4_Variants	CTGGTCGAGGAATCCTGGACGATT	3' ETS Variant
ACT2_F	TCGGTGGTTCCATTCTTGCTTC	RT-PCR
ACT2_R	CTGTGAACGATTCCTGGACCTG	RT-PCR

Supplementary Table 1 - Primer used in this study
