# Supplementary material for: Gene dosage compensation of rRNA transcript levels in Arabidopsis thaliana lines with reduced ribosomal gene copy number
Source: Plant Cell. 2021 Feb 2;33(4):1135–50. doi: 10.1093/plcell/koab020 (PMC8225240; doi:10.1093/plcell/koab020)
Supplement: koab020_Supplementary_Data [file koab020_supplementary_data.zip › SuppFig2.pdf]

Chromosome 4 duplication site

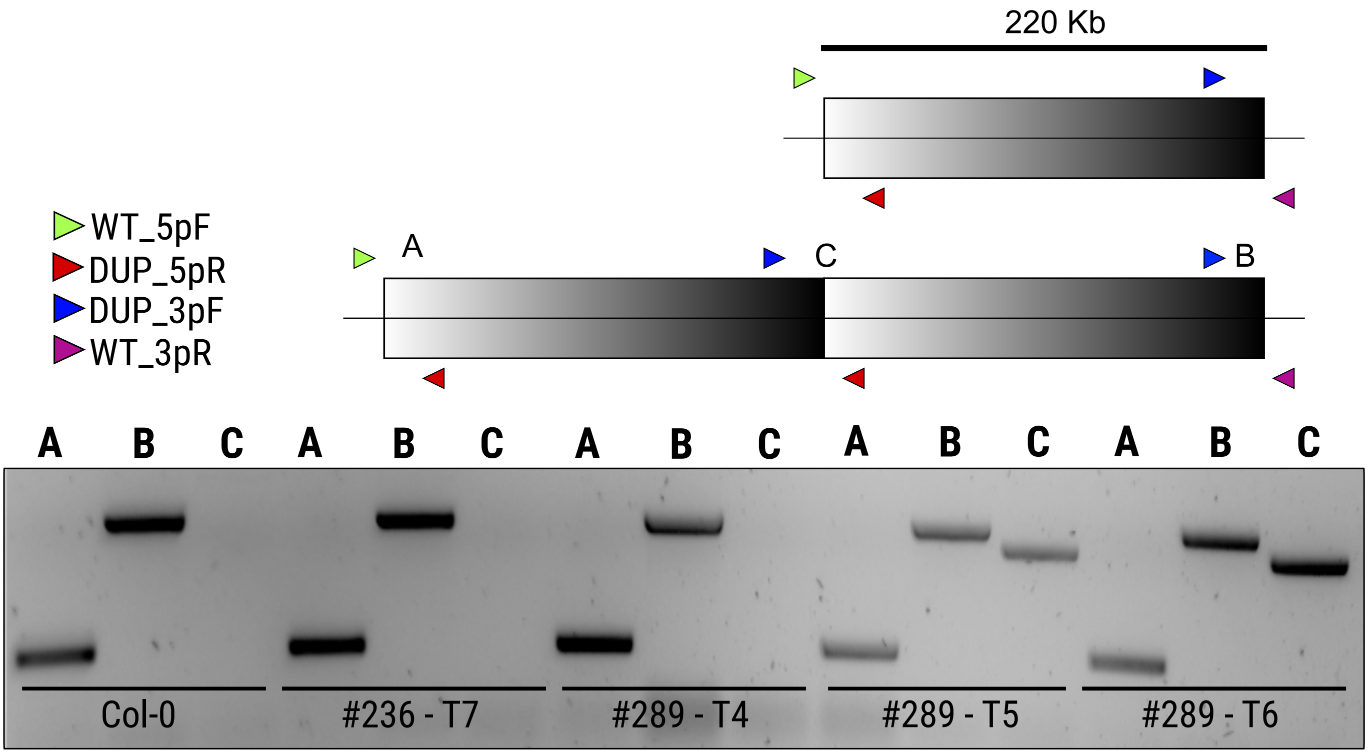

A - WT_5pF + DUP_5pR - 277 bp spanning 5' duplication site
B - DUP_3pF + WT_3pR - 950 bp spanning 3' duplication site
C - DUP_3pF + DUP_5pR - 784 bp tandem duplication

Supplementary Figure 2 – Identification of chromosome segment duplication in line #289. (Supports Figure 4). Using data retrieved from Nanopore sequencing, primers were generated along the duplication to test for presence of the duplication in different generations. While absent in T4 generation, chromosome 4 segment duplication appears in T5 generation and is present in the T6 generation analysed.
