# Supplementary material for: Gene dosage compensation of rRNA transcript levels in Arabidopsis thaliana lines with reduced ribosomal gene copy number
Source: Plant Cell. 2021 Feb 2;33(4):1135–50. doi: 10.1093/plcell/koab020 (PMC8225240; doi:10.1093/plcell/koab020)
Supplement: koab020_Supplementary_Data [file koab020_supplementary_data.zip › SuppFig1.pdf]

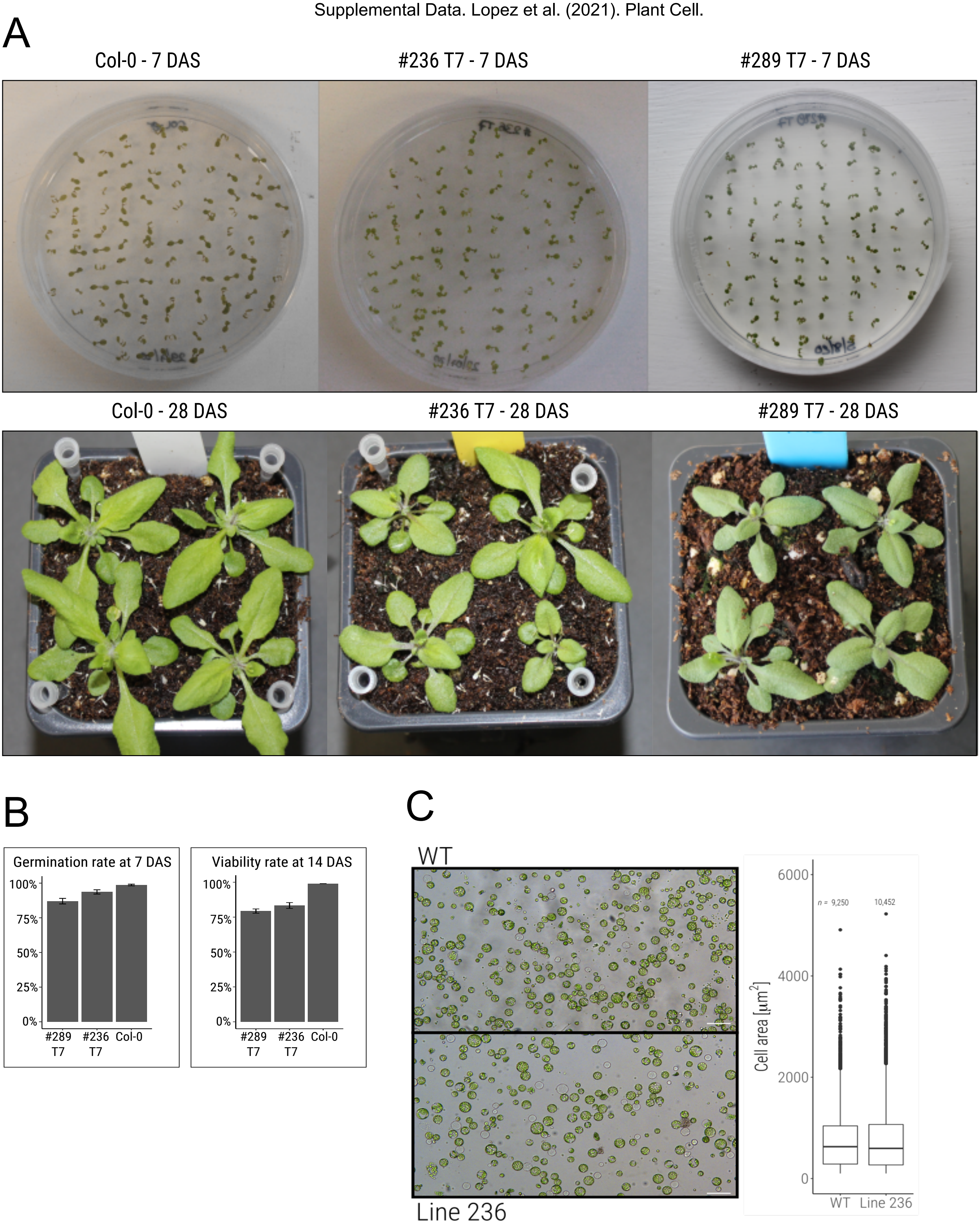

Supplementary Figure 1 - Plant viability and cell size in LCN lines. (Supports Figures 2 and 3) (A) WT and LCN plants at 5 days and 28 days after sowing (B) WT and LCN germination rates and plant viability (C) Representative images of mesophyll protoplasts in WT and #236 backgrounds. Measurement of cell area reveals a 6% decrease in line #236. (T test, p = 0.011, Scale bar = 100 µM).
