# Supplementary material for: Gene dosage compensation of rRNA transcript levels in Arabidopsis thaliana lines with reduced ribosomal gene copy number
Source: Plant Cell. 2021 Feb 2;33(4):1135–50. doi: 10.1093/plcell/koab020 (PMC8225240; doi:10.1093/plcell/koab020)
Supplement: koab020_Supplementary_Data [file koab020_supplementary_data.zip › tpc.00486.2020-s04.pdf]

# Gene dosage compensation of rRNA transcript levels in *Arabidopsis thaliana* lines with reduced ribosomal gene copy number

Francesca B. Lopez, Antoine Fort[a,b*], Luca Tadini[c], Aline V. Probst[d], Marcus McHale[a,b], James Friel[a], Peter Ryder[a], Frédéric Pontvianne[e], Paolo Pesaresi[c], Ronan Sulpice[b], Peter McKeown[a], Galina Brychkova[a], and Charles Spillane

*Corresponding author: Charles Spillane, charles.spillane@nuigalway.ie*

**Review timeline:**

| | | |
|---|---|---|
| **TPC2020-BR-00486** | Submission received: | June 24, 2020 |
| | 1st Decision: | July 27, 2020 *revision requested* |
| **TPC2020-BR-00486R1** | 1st Revision received: | Oct. 27, 2020 |
| | 2nd Decision: | Nov. 18, 2020 *accept with minor revision* |
| **TPC2020-BR-00486R2** | 2nd Revision received: | Dec. 18, 2020 |
| | 3rd Decision: | Dec. 24, 2020 *acceptance pending, sent to science editor* |
| | Final acceptance: | Jan. 18, 2021 |

**REPORT:** (The report shows the major requests for revision and author responses. Minor comments for revision and miscellaneous correspondence are not included. The original format may not be reflected in this compilation, but the reviewer comments and author responses are not edited, except to correct minor typographical or spelling errors that could be a source of ambiguity.)

**TPC2020-BR-00486 1st Editorial decision – *revision requested*** **July 27, 2020**

We have received reviews of your manuscript entitled "Homeostasis of rRNA levels in Arabidopsis thaliana populations with reduced ribosomal gene copy number." Thank you for submitting your best work to The Plant Cell. The editorial board agrees that the work you describe is substantive, falls within the scope of the journal, and may become acceptable for publication, pending revision and potential re-review.

We ask you to pay attention to the following points in preparing your revision: The reviewers found that the approach was an interesting and clever approach to address an important question. They do, however, identify a number of issues with the manuscript that suggest additional controls are needed. You can revise the manuscript to address these comments or provide rebuttal arguments to the critiques.

We also note that studies in maize of varying the dosage of the NOR have been conducted years ago and while the studies are old, they suggest some different responses. Lin (1955, Chromosoma 7: 340-370) showed that changing the dosage of the NOR had a dosage effect on the quantity of RNA within the NOR. This study was performed before the function of the NOR was even known but the results do suggest that there is no compensatory effect occurring under the different types of dosage manipulations studied. Further, Buescher et al. (1984, Biochemical Genetics 22: 923-930) found that a line with two NORs did not affect the amount of rRNA. You might compare your results to these classical studies.

----------------------------------------------------------------------- Reviewer comments:

[Reviewer comments shown below along with author responses]

**TPC2020-BR-00486R1 1st Revision received** **Oct. 27, 2020**

Reviewer comments and **author responses**:

Editors comments:

We also note that studies in maize of varying the dosage of the NOR have been conducted years ago and while the studies are old, they suggest some different responses. Lin (1955, Chromosoma 7: 340-370) showed that changing the dosage of the NOR had a dosage effect on the quantity of RNA within the NOR. This study was performed before the function of the NOR was even known but the results do suggest that there is no compensatory effect occurring under the different types of dosage manipulations studied. Further, Buescher et al. (1984, Biochemical Genetics 22: 923-930) found that a line with two NORs did not affect the amount of rRNA. You might compare your results to these classical studies.

**We have added these two studies and their relevance to the paper in the Discussion section (lines 484-491).**

Reviewer #1:

This manuscript focuses on the effect and mechanism of reduced rDNA copy number in Arabidopsis. The authors utilized crispr-cas9 system and obtained lines with reduced rDNA copy number to ~10% and found it does not affect the abundance and transcription rate of rRNA. They found a reduction of silencing histone markers at the rDNA loci, which could possibly lead to the dosage compensation of rDNA genes. Changes in global gene expression are also observed in LCN lines. It is an interesting topic. However, there are a few problems with the method being used or the conclusion being drawn. In addition, this manuscript contains numerous typos that need to be corrected.

**We are pleased that the reviewer considers the topic of our study to be of interest. We address the points raised below, in addition to having thoroughly proof-read the text of the revised manuscript.**

Major points:

1. The authors should provide data regarding the relative 45S CN from T1-T8, at least for the generations (e.g. T1, T4, T5, T6, T7) that were used in this study.

**This has now been carried out and the data added to Figure 2; the T8 generation was removed since it was not used in any further experiment.**

It would be good if the viability or % healthy plants of the above mentioned plants are also described.

**We have provided sample pictures of the seedlings at 7DAS and plants at 28 DAS, as well as data quantifying the viability of plants at the T7 generation for lines 236 and 289 (Supplementary Fig 1).**

It is interesting to know if there is any variation. The authors mentioned they were trying to find the lowest 45S CN threshold. Therefore, Is T9 not viable?

**Preliminary analysis of T9 show that it is viable. The word "threshold" was perhaps inadvertently misused: we intended to communicate that, despite the continued presence of Cas9 activity in the egg cell, rDNA Copy Number appears to level out at ~10% in the T6/T7 generations. This has now been clarified in our discussion, lines 327-330.**

2. The model in Figure 5 is mainly supported by the data presented in Figure 2. However, it is not well supported that NOR2 are transcriptionally competent with the current evidence.

**As described above, we apologise for miswording this section. We clarify that the term "transcriptionally competent" means only that NOR2 appears to be available for transcription on the basis of its nuclear location. Our intention was not to claim that NOR2 is actively transcribed in rosette leaves, simply that (since it localises at the nucleolus) it appears to be available for transcription. In WT rosette leaves for example, NOR4 is available for transcription throughout vegetative development, however not all rRNA genes present on NOR4 are actively transcribed. This can be also inferred from the large round signals of NOR4 in WT which indicates that a large number of rDNA genes on NOR4 are condensed into large chromocentres flanking the nucleolus.**

We also note that in our FISH experiments, a certain level of chromatin condensation is still visible in all four NORs (i.e. larger dotted signals), indicating that a level of chromatin condensation is still present.

**These points have been clarified at lines 185-189.First, L208: The authors may need to elaborate how they draw this conclusion that NOR2 remains functional based in Figure 2C (also did not mention what kind of blot this is).**

**The blot in figure 2C is an agarose gel electrophoresis following RT-PCR, we address these points in lines 213-220.**

**Level of VAR1 is different across lines and not correlated with CN in cDNA 5 DAS.**

**As the variant assay has changed we have addressed these points in lines 185-189**

Second, figure 2D: The authors need to provide evidence that their results of WT and #236 are comparable in their imaging analysis. There is no scale bar.

**These have now been added.**

Meanwhile, it is not clear whether these FISH results are performed simultaneously, using the same exposure time for imaging, etc.

**We confirm that FISH experiments were performed simultaneously on 5 DAS seedlings and 20 DAS rosette leaves using identical conditions for hybridisation and imaging; this is now clarified in the Methods (line 675).**

Is their explanation for the shape of nuclei being nor round? (compared with Picart-Picolo et al, 2020 & Pavlištová et al., 2016).

**We hypothesise that nuclei are not round in rosette leaves due to aberrant leaf and cell shape but as we have yet to perform a quantitative analysis of the various phenotypes observed, we prefer not to speculate. We would also like to point out that rarely leaf nuclei are round due to the presence of a large vacuole. The elongated shape of seedling nuclei is instead likely due to the use of whole seedling, including roots and stems in which the nuclei are also elongated in wild-type plants.**

Meanwhile, the blurry signal in 2D is not sufficient to prove NOR2 and NOR4 are both actively transcribed.

**As noted above, we do not wish to claim this and have clarified our text accordingly (see response above)**

Third, why VAR1 is not detected in #236 in rosette? In figure legend 2, the authors stated 'It appears however that VAR1 associated with NOR2 is still largely silenced during development.' And VAR3's level is relative similar between WT and #236. It seems to conflict with the proposed model.

**We agree with this: we carried out the RT-PCR assay on variants using the T7 generation to correlate it with FISH experiment (line 185).**

3. Figure 3C. Usually the RNA spikes are added to a fixed amount of RNA samples rather than diluted tissue as described in material and methods. Is there a specific reason why? Otherwise the result could be inaccurate given that the RNA extraction efficiency could be variable in different RNA extractions.

**Since a fixed amount of spike is added to a known quantity of biomass, the amount of spike recovered after the RNA extraction is proportional to the amount of RNA in the biomass, allowing for a direct comparison of the number of molecules per unit of biomass. Of course, as reviewer 1 points out, if the biomass in one sample was not ground appropriately the RNA extraction itself may not be representative of the sample. However, all samples were treated in the same manner and the biological replicates (n=3) indicate an accurate mean for the two genotypes. This method was successfully employed in several previous studies (Pal et al. 2013; Ishihara et al. 2017; Piques et al. 2009; Vaid et al. 2020) which we now cite (line 636). Furthermore, adding the spikes to a fixed amount of RNA rather than biomass could give erroneous results in the case of a differential expression of ribosomal RNAs, which was the question investigated here. Indeed, since rRNAs represent the majority of total RNAs in a cell, normalizing by the amount of total RNA (as is done in northern blots) could potentially erase the possible intrinsic difference in rRNA levels between samples, leading to the risk of false negative results. This precision was added to the materials and methods section (lines 562-566).**

4. Is there a difference in the nucleolar size in the LCN lines compared with the control as described in some publications.

**We did not observe any significant difference in nucleolar size.**

How about cell size?

**We could speculate that cell size might have been affected by the reduction of genomic DNA content, as correlation**

**between eukaryotic cell size and genome size have been previously proposed (Beaulieu et al., 2008), however this may be the focus of future work.**

The authors reported global gene expression changes, does it affect the size of the transcriptome? This needs to be addressed.

**Estimating transcriptome size would require a transcriptomic analysis based on a known number of cells, for which their effective ploidy level would also need to be determined. Since our transcriptomic analysis is based on whole seedlings, the experimental design did not allow for an estimation of the transcriptome size. However, in our reanalysis involving a fold change cutoff (lines 293-317), we found a significantly lower amount of gene expression changes (~570 versus ~6,000). Hence, the impact of rDNA copy number reduction is likely less global as we previously thought, and only seems to impact a limited number of biological pathways. Therefore, it is unlikely that transcriptome size plays a role in the gene expression changes observed here. However, estimating the effective ploidy (e.g. endoreduplication levels) and the cell size of plants with low rDNA copy number would certainly be interesting follow-up experiments to determine possible physiological/developmental effects.**

5. Is expression of chromatin remodelling factors changed in LCN lines? This would be interesting to investigate.

**Indeed, changes in the expression of chromatin remodelling factors is an interesting question. However, chromatin modification is included in the Mapman analysis and we found no evidence for a general deregulation of genes involved in these pathways. It is possible that if deregulation of some key chromatin remodelling genes occurs, it happens at later stages of cellular differentiation, i.e. in rosette leaves or during floral development. However we think this may fall beyond the scope of this manuscript.**

6. Figure 3B & 3D. Is that only one plant of each condition was used for northern blot? The authors should provide biological reps or also include #289 for this type of analysis.

**We agree with these points and have now added the results of line #289, which confirm the conclusion.**

7. Are the enriched bins for up-regulated DEGs different from down-regulated DEGs?

**We have significantly modified the transcriptome analysis for this revised manuscript. We have now applied a fold change cut-off (1.5 fold change compared with wild type) for the detection of DEGs. This change was made to reduce the impact of confounding batch effects from a common cultivation environment within each genotype on the analysis of the effect of genotype.**

**Upon reflection we consider that this is necessary as a large number of DEG's were resolved as statistically significant despite displaying very small fold changes and poor agreement in the responses of these genes between LCN lines (24% of common genes were antagonistic in their expression levels in these lines.).**

**Our revised analysis now detects ~570 DEGs, of which ~50% are dysregulated in both lines with ~85% of these in the same direction. We then performed the same mapman bin enrichment analysis but on the subset of genes up and down- regulated, as Reviewer 1 suggested (Figure 4B and Figure 4E). This Up/Down analysis on fold-change selected transcripts shows twelve enriched bins, which are part of four main biological functions: Amino acid biosynthesis, Cell wall organisation, Enzyme classification and Secondary metabolism. Biosynthesis of pyruvate family amino acids, as well as glucosinolate biosynthesis and degradation were up-regulated in both LCN lines, while oxydorecuctase enzymes were down-regulated.. Finally, we found that the dysregulation of cell wall genes was not consistent between the LCN lines (up-regulated in #236 and down-regulated in #289, hence is less likely to be a specific response to the reduction in rDNA copy number. We have modified the manuscript accordingly (lines 311-338).**

8. L522. Are the DEGs in the tandem duplication in #289 affected in the same or different manner compared to #236?

**We have added the results of line 236 to Figure 4C to answer Reviewer 1's comment. Of the duplicated region in line #289 (51 genes), line #236 displays two significantly deregulated genes (down-regulated), as opposed to the 18 up-regulated genes in line 289. Furthermore, the mean fold change of genes in this locus is 1.5 and 0.93 in lines #289 and #236, respectively. Hence, the DEGs identified in this locus in line #289 are likely a direct result of the tandem duplication. We have added the comparison with line #236 in Figure 4C and the text to best reflect this comparison and strengthen this conclusion (lines 312-317).**

9. The authors need to deposit the sequencing files.

**We have added the bioproject ID of the sequencing files, available in Sequence Read Archive. We thank the reviewer for the reminder!**

10. It would be better if the authors provide more background information regarding VAR1-4. For example, which region in Figure 1A is varied in VAR1-4. How are they different?

**We have added a schematic diagram of the 3’ rDNA variants in Figure 2, as previously elucidated by (Pontvianne et al., 2010)**

Minor points & typos:

1. L188 "150%" seems to conflict with L191 "160%". L191 160% conflicts with L201 150% and L208 140%. Are they from different generations?

**The correct value was 160% - the typos in the others have been corrected.**

2.L199 "45S 45S rDNA"

**Corrected**

3.L206. VAT1 expression seems to be not as weak as described in Figure 2C "5 DAS".

**Comment is unclear as VAR1 is always expressed in seedlings of WT and LCN.**

4.L206. Is '< 15 DAS' supposed to be 5 DAS? Or is rosette actually 15 DAS?

**15 DAS indeed indicates rosette leaf (now clarified).**

5.Figure 2D. Why did the authors used < 7 DAS and > 15 DAS rather than 5 and 20 DAS.

**This was an incorrect annotation: the correct annotations are 5 DAS and 20 DAS and the figure has been corrected accordingly.**

6.Figure 3. In the legend, figure 3C and 3B are switched. No information regarding the statistical test in 3C.

**Corrected**

7.Figure 3E. Y-axis titles needs to be revised (not relative to H3). What does the error bar represent?

**Corrected – error bar represents standard error among three biological replicates (now specified in the figure legend).**

8.L286. should be 3E.

**Corrected**

9.Figure 4A. Is the figure legend 'Log2 FC' or just FC? The authors did not mention anything about taking log of fold change in the manuscript. What do the blue triangles represent?

**The results from the Wald test of the Sleuth analysis are expressed in log2 by default. We have added this precision to the materials and methods (lines 674-675).**

10.Figure 4E does not have a figure legend for genotype.

**We have added the figure legend of Figure 4E**

11.L328. "c."

**Corrected**

12.L377. "%%"

**Corrected**

13.L618-624. Information regarding northern blots are duplicated.

**Corrected**

14. Some of the references are duplicated.

**We have now corrected this**

15. L726. "Primers table"?

**We have added the table of primers in Supplementary Table**

Reviewer #2:

Wild type Col-0 *Arabidopsis thaliana* is estimated to have ~ 1,500 rRNA genes per diploid genome which are arranged in tandem repeats on chromosomes 2 and chromosome 4 in nucleolar organizer regions (NOR) referred to as NOR2 and NOR4. During early stages, rRNA genes of both NORs are expressed, however, over the course of development, rRNA genes located on NOR2 are selectively silenced, whereas rRNA genes on NOR4 remain active and are expressed. While the plant does not require all of its rRNA genes to be active, it is unclear what the minimum number of genes required for survival might be.

The manuscript entitled "Homeostasis of rRNA levels in Arabidopsis thaliana populations with reduced ribosomal gene copy number" addresses the question "What is the biological consequence of having a low copy number of rRNA genes?". The authors creatively use Crispr-Cas9 technique to create Low Copy Number (LCN) lines with as few as 10% of the original rRNA gene copies. Interestingly, in these LCN lines, the rRNA transcriptional rates and steady-state levels are very similar to that of wild type. The authors find that this may be due to loss of the silencing histone modification H3K9me2 leading to both NORs being active which may compensate for the low copy number. This conclusion is well supported by rRNA copy number quantification, 45S FISH, H3K9me2, and H3K9Ac chromatin immunoprecipitation. The authors also observed a chromosome segmental duplication, indicating possible genome instability associated with low rRNA copy number. Widespread gene expression changes in these lines also indicate possible compensation of rRNA gene loss.

This works presents an interesting new system that could be useful in understanding minimum 45S copy number, DNA repair, NOR silencing mechanisms, global chromatin regulation, etc. In addition, this work challenges the dogma that developmentally silenced 45S copies are retained in order to provide sufficient rRNA transcription during early development.

**We thank the reviewer for their comments and are pleased that they consider our work to provide an interesting new system for future study.**

Major points throughout:

• Clarify which generation (T1,T4,T7) each experiment is using every time the plant line is mentioned in the results section (line #236 T4 vs line #236) or clearly label it on each subfigure (not figure legend or methods). It appears that across various experiments T1,T4,T5,T6,T7, and T8 are all used. Figure 3 is particularly unclear on this.

We have added this information to our revised figures, legends and manuscript.

**To summarise:**

- **FISH and Variant were performed on plants of the T7 generation**
- **RNAseq was performed on the T7**
- **Nanopore sequencing was done on T6 (#236) and T5 (#289)**
- **ChIP was done on T7 generation**
- **Northern blots and run-on were done on T7 generation**
- **rRNA quantification with spikes was done on T4 generation**

The authors mention ~375 copies of the 45S per haploid genome several times (lines 124, 358). This is half of the estimated copy number in Col-0 from previous work, as each NOR was estimated by Copenhaver et al. to have ~375 copies per haploid genome. This factors into their conclusion on lines 362-367, where the authors state that mutants with ~7% of WT copy number have about 25 copies of the 45S. They then compare this number to S. cerevisiae minimal 45S numbers which are estimated at 20. However, 7% of ~750 is closer to 52 copies, which is not as similar to S. cerevisiae.

**This was an accidental oversight on our part, and we thank the reviewer for their comment. We have revised this in the MS.**

The authors comment several times that most LCN line individuals appear phenotypically normal. They should add an image of the LCN lines compared to WT Col-0 at similar growth times, especially considering that the transcriptomic analysis revealed widespread DEGs.

**We have added images of the LCN lines across different generations at 7 DAS and 28 DAS (Supplementary Figure 1).**

The authors regularly make the point that the reduction in copy number in LCN lines is causative to reduction in H3K9me2, nuclear organization, etc. It may also be that loss of H3K9me2 is merely a reflection of gene activation and is correlative, not causative. It is also not clear whether simply cutting the 45S regularly with Cas9 might cause chromatin modification changes, regardless of the rDNA copy number. To clearly test this, this study needs a control line in which the same Cas9 construct does not reduce copy number or increases it (like many of the transformant lines in Fig2A). Otherwise, the authors should emphasize that copy number reduction is only speculated to be causative of other changes. Copy number reduction is the simplest explanation, but it seems plausible that regular cutting by Cas9 (even limited to the egg cell) and subsequent repair could directly cause changes to chromatin marks and NOR location.

**We agree with Reviewer 2, while it seems expected that silencing marks are less enriched due to the reduction in copy number, we agree that without a secondary control it is not possible to declare a causative mechanism. This point is now clarified in our Discussion at line 280.**

Figure 1C. deletion/insertion images are not clear; they don't keep the same copies visible between each 'before and after' cartoon.

**This has now been fixed.**

Figure 2B. The scale could be adjusted so it is easier to view where the bars line up

**This has now been fixed.**

2B. If these are close to 'minimal copy number' lines, are the authors seeing the low copy numbers plateau over several generations? Is T6 or T7 showing as low numbers as T8? If not, would later generations get lower numbers?

**We observed CN to stabilise between 20% and 10% in T5/T6/T7 generations, and for this reason we suggest that the minimal functional CN may be ~10% under the growth conditions used.**

2C. The use of T4 in 2C and T7 in 2D seems to have conflicting results. The authors state that both NORs are active based on the FISH results in 2D, however, not all of the rRNA variants are expressed in the rosette in 2C. It would be good to see a comparison of RT results between T4 and T7. Additionally, if all rRNA copies are indeed active in the LCN lines, there should not be a big difference in the ratio between the different VARs in gDNA and cDNA. The authors also state that VAR1 is silenced in all the lines they tested but that is not the case for line #52 where VAR1 is still expressed in the rosette.

**We agree with Reviewer 2, for this reason the variant experiment was done on T7 to provide a full correlation with the results observed in the FISH, indeed we observed that VAR1 is expressed in rosette leaves of both LCN.**

**With regard to the difference in the ratio of cDNA and gDNA we would like to stress that the assay is meant to be qualitative not quantitative. This RT-PCR only gives a relative idea of the expression levels between variants.**

**These points are now clarified at lines 185-189**

2C. There is no mention of the RT protocol for the rRNA variant analysis under the results or the methods section. Were seedlings/leaves pooled for the RT?

**This has now been added and the pooling strategy clarified, see lines 607-619**

Figure 3B and 3C legends are switched

**This has now been corrected**

Figure 4 A small summary of nanopore sequencing data (genome coverage, read N50 for each run) is necessary in either the figure or results section.

**We have added a summary of the nanopore sequencing including total number of reads, mapping percentage, mean coverage and mean read length to the manuscript (Table 1).**

Line 295 calls this section 'de novo assembly', but there is no suggestion of a de novo assembly being performed in either this section or the methods. Figure 4A appears to be reference based mapping according to the methods.

**Indeed, this was a mistake on our part. The analysis is indeed the results of a reference-based mapping. We have corrected the manuscript accordingly.**

Methods

Nanopore sequencing needs to include what basecalling software and version were used.

**We have added this information to the manuscript.**

There is no description of the PCR or RT-PCR used in 2C.

**Fixed**

Minor points:

• Line 55, "hundreds or thousands of rRNA copies" - should probably be "hundreds or thousands of rRNA gene copies".

**Corrected**

• Line 383: the authors make the point that loss of CN occurs randomly, however with VARs from only 3 lines examined, the sample size is too low to conclude this.

**Because of sequence identity of the 18S gene across both NORs, we suggest that it is very plausible that mutagenesis and insertion/deletions in the of 18S gene occurs randomly across both NORs although we agree with the point regarding sample size.**

In the Methods section, under "Northern blot and nuclear run-on assay" on page 21, lines 621-622 are a duplicate of lines 619-620.

**Corrected**

In the Methods section, under Chromatin Immunoprecipitation, line 728 regarding the normalization to HKX1 and Ta3 appears to conflict with the figure legend.

**Corrected**

<u>Reviewer #3:</u>

Lopez and colleagues investigate how homeostasis of rRNA production is achieved in lines that have severely compromised rDNA copy numbers. To that end, they have randomly depleted rRNA genes with a Cas9 approach, and successfully retrieved viable lines with dramatic copy number reductions. Based on their selection approach -for viable lines- they may well have reached the absolute lower limit of necessary rDNA copies that an A. thaliana line needs to survive. Although they only analysed a single Cas9-derived lineage for the main point of their study, they

conducted independent rRNA expression experiments and monitored two chromatin marks to support their main finding: rRNA transcriptional rates and steady-state levels remained the same as wild type plants.

In the process of analysing low rDNA copy number lines, they have identified signs of genomic instability in one of the Cas9-derived lines that appear to be a side-effect of reduced rDNA copy number. Although not the main objective of the study, this latter finding is relevant in the context of a recently published pre-print (Picart-Picolo et al., 2020 BioRxiv, which the authors properly cite and discuss) that also reported the emergence of chromosomal tandem duplications in low rDNA copy number lines in a more problematic background than the present study's Cas9-derived lines.

My two main concerns with this study are that the Results section corresponding to Figure 2 contains contradictory statements (see below), and that it appears the Cas9 construct was not segregated out after T1 in which they have already produced lines with "minimal" rDNA copy numbers.

**We would like to thank Reviewer 3 for their insightful analysis of our work and their comments. We have addressed these, to the best of our ability, by mainly eliminating the discrepancies among generations used for different experiments, as well as addressing why our experimental approach aimed to maintain Cas9 activity in the Egg Cell in each generation. We are also interested in comparing the effects we describe here in lines with comparable CN in a clean, Cas9-free background.**

Major comments

1. The first section of Results, when it came to the analysis of VARs, was highly confusing. I had to read it twice and finally was able to parse seemingly contradictory statements:

- On the one hand:

In Line #236 VAR4 is highly expressed in T4, meaning NOR4 is active.

In Line #236 VAR1, though abundant at the DNA level, is weakly expressed (or completely silent) in T4, meaning NOR2 is inactive.

- On the other hand:

In Line #236 both NOR signals "localize inside the nucleolus" during development in T7, "suggesting that the 45S rDNA gene copies of NOR2 remain active" (text lines 239-240).

Is this different outcome for line #236 due to the different generations (T4 and T7) in which this line was analysed for the experiments in Figures 2C and 2D? Could it be that discrepancy is due to an alteration in 45S rDNA CN -or configuration, see below point about Cas9- between both generations? It would be advisable to add CN information for line #236 at the generations in which the experiments are conducted, or alternatively, present both kinds of experiments in the same generation.

**We apologise for the confusing wording in the previous version of our manuscript and agree with R3 regarding the possible contradiction. To clarify this, we carried out the variant analysis on plants of the T7 generation to avoid discrepancies with FISH result. In this case, the variant analysis shows that in lines #236 and #289 VAR1 also remains active throughout vegetative development.**

**This section has now been rewritten to avoid ambiguity (lines 185-189) and the additional CN information provided.**

- Also confusing, in lines 222-231, how do the authors know that in their experiment the two foci that localize at the nucleolus are NOR4 in Figure 2D? I understand from the literature on Col-0 that this is often the case for that particular strain; however, the author's own data on Col-0 WT (Figure 2C; forth panel from top to bottom for WT), seemingly contradicts that assertion, at least expressed so categorically. It seems as if their WT line keeps showing expression of NOR2 in rosettes as manifested by VAR1, thus it is expected that in the FISH data NOR2 would also be sometimes localizing at the nucleolus.

In addition, in text-line 208-209 it is stated that the inactivation of NOR2 also applies for line #52, but that doesn't seem to be the case according to Figure 2C (forth panel).

**We thank the reviewer for this comment, we have amended our Variant analysis to include the lines described throughout the manuscript. The new variant assay shows that in both LCN lines, VAR 1 appears actively transcribed during vegetative development in rosette leaves.**

2. It is unclear from only reading the methods section whether the authors left the construct with Cas9 in the genome during subsequent generations or if they segregated it out after T1. From the fact that they identified the insertion sites with Nanopore sequencing in T5 and T6, I understood it is the former.

If that were the case, the authors should elaborate on why this approach was necessary. I suspect it is because the authors wanted to guarantee their analyses were conducted in lines with a "stable" minimum of rDNA copies -indeed, Figure 2B is reassuring in that at T8 there was little CN variation. However, one would expect that all lines are subjected to Cas9-mutagenesis each generation. As a consequence, not only rDNA CN could vary from generation to generation, but also cluster configuration (i.e., NHEJ between rDNA clusters from different chromosomal origin to repair Cas9-induced DSB).

In light of the above, the analyses at T4 and T7 generations of those lines (Figures 2c and 2D) would also need a confirmatory rDNA CN assessment.

**Reviewer 3 correctly points out that we did leave the Cas9 construct in the genome following the original transformation. Our aim was to lower the number of copies as much as biologically possible, and that this might not be achievable within a single generation. Hence, we maintained the two low CN lines containing the transgene for several generations. We did not find major differences from generation to generation after the T4. We found a decrease of CN from the T1 (~30%) to the T4 (~15%), which remained stable afterwards. Hence, the T4 and T7 generations used here effectively have the same copy number, and explaining our conclusion that ~15% CN is likely the minimum achievable 45S CN in Arabidopsis. We have added the copy number results over the different generations to the manuscript. As Reviewer 3 points out, future studies will indeed focus on transgene-free low CN lines to further characterise the impact(s) of copy number reduction on plant phenotype and genome.**

Minor comments

1. When did the tandem duplication in Chr4 appear? The authors detected it with Nanopore sequencing in T5 or T6. PCR of the novel junction derived from the duplication event would be simple enough for the authors to identify in which generation the event took place. Hopefully not as a by-product of the original transformation, but later on, which would give stronger support to the hypothesis that it emerged as a consequence of genome instability due to low rDNA copy number.

**We added a supplementary figure to show that the duplication appeared in T5 generation.**

2. In line 358 of the discussion, the authors claimed ~375 rDNA copies per haploid genome in Col-0. Where did that estimate come from? That estimate seems at the lowest end of what has been reported in the literature. In Ler-0 for instance, Copenhaver and Pikaard (1996) estimated 350-400 copies at each rDNA cluster, and Col-0 might not be that different. Nevertheless, from the methods section, it seems that the authors employed a single copy gene for the qPCR CN quantification. The authors could well use their own Col-0 WT estimate for the discussion -in case they are not already doing so.

**We noted that this was a typo, we meant ~375 copies per NOR. Indeed, we use our own Col-0 WT for all qPCR quantifications.**

3. Figure 1C. Please add the explanation for the colour code (gray vs. white). I am assuming the authors want to differentiate positively cleaved copies from those that were spared, but that has to be spelled out.

**We amended the figure legend to better explain the colour coding.**

4. Legends for Figure 3B and 3C appear to be swapped.

**Corrected**

5. Figure 4A. Only until the Discussion one understands what do the insertion diagrams mean (Cas9 insertion sites). Please add them to the figure legend. Also, unclear what the sequential color code in the rectangle outside of each chromosome means. If it does not convey useful information, better remove it.

**We have removed the colour codes for the chromosomes, and added a clearer legend/annotation for the Cas9 transgene insertion sites.**

6. "Our approach" in line 531.

**Corrected in revision.**

**TPC2020-BR-00486R1 2nd Editorial decision – *accept with minor revision* Nov. 18, 2020**

We have received reviews of your manuscript entitled "Gene dosage compensation of rRNA transcript levels in Arabidopsis thaliana populations with reduced ribosomal gene copy number." On the basis of the advice received, the board of reviewing editors would like to accept your manuscript for publication in The Plant Cell. This acceptance is contingent on revision based on the comments of our reviewers. In particular, please consider the following: Reviewer #1 provides a scenario of how the rRNA transcriptome size might not be assayed correctly. This should be able to be addressed easily by comparing the cell size in the LCN and normal lines. Both reviewers have some minor issues to correct.

Please highlight all changes and include a detailed annotation of changes of the text, with line numbers, and noting your responses to the comments.

Note from the Editor in Chief: we are making an effort to reduce the use of red-green color comparisons as these are not easily distinguished by individuals with color vision deficiencies. I noticed that Figure 5 uses red and green highlights; perhaps you can change this to magenta instead of red, or use other colors.

**TPC2020-BR-00486R2 2nd Revision received Dec. 18, 2020**

Reviewer comments and **author responses**:

Reviewer #1:

The authors have solved most of my former concerns. However, my major concern for this manuscript is that the authors may overlook the effect of changes in the size of rRNA transcriptome in measuring the rRNA accumulation.

1. The measurement of rRNA molecule abundance in Figure 3B and Figure 3C are relative to total RNA or per biomass (not per cell). Although it was claimed in Figure 3C that the spikes were added per biomass, this in nature assumes the RNA extraction efficiency per sample equals. Therefore, Figure 3C is relatively 'equal' to rRNA per total RNA being extracted from equal amount of biomass. The assumption to be tested in Figure 3B-C is whether the rRNA accumulation changes in LCNs. If we assume reduced rDNA copy leads to reduced rRNA transcripts, it would lead to reduced transcriptome size (total RNA) per cell, which would lead to reduced cell size. In this case, no matter normalized to the equal amount of total RNA (like in Figure 3B) or the equal amount of biomass (Figure 3C), it does not measure rRNA copy per cell. For example, in an extreme hypothetical condition, if we assume in the WT, there are 4 cells each with 2000 rRNA molecules; whereas in LCNs, the same amount of total RNA or biomass would contain 8 cells each with 1000 rRNA copy (assuming LCN's cell size is half of the WT and mRNA transcriptome is very small compared to rRNA transcriptome). Using neither northern blot nor qPCR relative to spikes would reveal the rRNA copy variation per cell in this case. Therefore, I think the authors' conclusion could be better supported if they could do a transcriptome size or cell size measurement or calculate rRNA molecules per genome. I disagree with the author's response that 'estimating transcriptome size would require a transcriptomic analysis based on a known number of cells' (see Coate and Doyle, 2010). Furthermore, the authors responded that there are relatively small changes in gene expression reflected by the number of DEGs. However, this only reflects the mRNA

transcriptome size. It does not reflect the rRNA transcriptome nor the total RNA transcriptome consider the mRNA-seq used mRNA-enrichment in the library preparation (presumably polyA selection).

**We agree with Reviewer 1 that if cell size was dramatically affected by loss in rDNA copy number, a normalization by dry weight might not be representative of the number of cells / genomes in the plant. To investigate this, we measured the cell size of WT and low rDNA copy number plants and found a small difference of ~6% between low CN and wild type plants. Given such a small difference in cell size, we do not expect a major difference in the number of cells in 1 mg of WT or low CN plants. Hence, our spike assay is likely an accurate biological representation of rRNA accumulation in WT and low CN plants – and indicates the presence of a strong gene-dosage compensation mechanism. Given these findings we do not expect significant differences in transcriptome sizes.**

2. It is unclear what is the major point for figure 2C. The authors need to demonstrate what the % above the middle panel refer to. Are they trying to demonstrate different LCNs have different expression patterns of VARs? It would be better if this could be better organized. If a qRT-PCR rather than RT-PCR is performed, maybe it could better support Figure 2D.

**The percentage in Figure 2C is the % 45S Relative Copy Number, it has now been re-worded in the figure legend as it might have been misleading (Lines 790-791) Specifically, the % of relative 45S copy number was calculated by qPCR using the same DNA sample used to amplify the 3' variants by RT-PCR.**

**The major point of Figure 2C is to support our conclusion that NOR2 is available for transcription The presence of a band corresponding to VAR1 (present only on NOR2) in RNA extracted from 28 days old plant indicates that it is actively transcribed in line #289 and #236 compared to WT, where this variant is silenced.**

**Further, 45S rDNA variants in Arabidopsis can only be characterized by RT-PCR as the same primers are used that result in different band sizes depending on the variant (Pontvianne et al., 2010; Durut et al., 2014; Pavlištová et al., 2016; Pontvianne et al., 2012).**

3. Consider the cas9 still remains in the system and that the manuscript lacks a control with the cas9 components but not decreased rDNA copy number, it is not convincing that ~570 DEGs are due to reduced rDNA copy or nucleolus organization.

**We would like to thank Reviewer #1 for their comment and assure them we are working towards answering these further questions. Regarding the present study, while the cas9 transgene is indeed still present in our low CN lines, its promoter is restricted to the egg cell. Hence, the presence of the transgene is unlikely to impact gene expression at seedling stage. Nonetheless, future studies will focus on the precise characterisation of genomic, transcriptomic and possible phenotypic/environmental responses changes in low CN lines that do not contain the cas9 transgene.**

Minor points:

1.Did not find Vaid 2020 in the reference (from the author's response)

**Reviewed and corrected (Line 561)**

2. Figure 3 legend. 'otherribosomal'.

**Reviewed and corrected**

3. Figure 4. It is unclear why the fold change of gene expression is in log2 in Figure 4A, but not in log2 in Figure 4C. The brackets are not directly pointing to the duplications/deletions in Figure 4A.

**Figure 4C expression results were indeed in log2, we have now added this detail to the figure. We have also adjusted the brackets in Figure 4C and added the precise location of the duplication.**

<u>Reviewer #3:</u>

I have previously reviewed the manuscript by Lopez et al. submitted to The Plant Cell journal.

To my concern regarding the contradictory statements from the previous version of Figure 2, the authors have opted for displaying the results of their analyses in lines from T7 only. They now show that "both NORs are available for transcription" in lines #236 and #289.

As for my inquiry of why to maintain Cas9 activity in the Egg Cell in each generation, the authors have satisfactorily addressed it in the discussion.

The rest of my minor comments were also corrected.

I have previously overseen that the title could be slightly misleading in the "Arabidopsis thaliana populations" part. Although not explicitly referring to natural populations, it might give that impression to readers. "Arabidopsis thaliana lines" would be my suggestion, but I leave it up to the authors.

> **We would like to thank Reviewer 3 for their contribution and for adding value to this work. The authors also agree the word populations could mislead the reader; hence the title is now changed to contain the word lines instead of populations.**

Minor change: Please correct typo in Line 460: "likely to play a role role"

> **Reviewed and corrected**

**References**

**Durut, N. et al.** (2014). A Duplicated NUCLEOLIN Gene with Antagonistic Activity Is Required for Chromatin Organization of Silent 45S rDNA in Arabidopsis. Plant Cell **26**: 1330–1344.

**Pavlištová, V., Dvořáčková, M., Jež, M., Mozgová, I., Mokroš, P., and Fajkus, J.** (2016). Phenotypic reversion in fas mutants of Arabidopsis thaliana by reintroduction of FAS genes: variable recovery of telomeres with major spatial rearrangements and transcriptional reprogramming of 45S rDNA genes. Plant J. **88**: 411–424.

**Pontvianne, F. et al.** (2010). Nucleolin is required for DNA methylation state and the expression of rRNA gene variants in Arabidopsis thaliana. PLoS Genet. **6**: 1–13.

**Pontvianne, F., Blevins, T., Chandrasekhara, C., Feng, W., Stroud, H., Jacobsen, S.E., Michaels, S.D., and Pikaard, C.S.** (2012). Histone methyltransferases regulating rRNA gene dose and dosage control in Arabidopsis. Genes Dev. **26**: 945–957.

**TPC2020-BR-00486R2 3rd Editorial decision – *acceptance pending*** **Dec. 24, 2020**

We are pleased to inform you that your paper entitled "Gene dosage compensation of rRNA transcript levels in Arabidopsis thaliana lines with reduced ribosomal gene copy number" has been accepted for publication in The Plant Cell, pending a final minor editorial review by journal staff.

**Final acceptance from Science Editor** **Jan. 18, 2021**
